# Supplementary material for: Perinatal Mental Illness in the Middle East and North Africa Region—A Systematic Overview
Source: Int J Environ Res Public Health. 2020 Jul 29;17(15):5487. doi: 10.3390/ijerph17155487 (PMC7432515; doi:10.3390/ijerph17155487)
Supplement: Supplementary file 1 [file ijerph-17-05487-s001.pdf]

Supplementary Material

Table S1. PRISMA 2009 Checklist [1].

Section/topic	#	Checklist item	Reported on page #
**TITLE**			
Title	1	Identify the report as a systematic review, meta-analysis, or both.	1
**ABSTRACT**			
Structured summary	2	Provide a structured summary including, as applicable: background; objectives; data sources; study eligibility criteria, participants, and interventions; study appraisal and synthesis methods; results; limitations; conclusions and implications of key findings; systematic review registration number.	1
**INTRODUCTION**			
Rationale	3	Describe the rationale for the review in the context of what is already known.	2-3
Objectives	4	Provide an explicit statement of questions being addressed with reference to participants, interventions, comparisons, outcomes, and study design (PICOS).	3
**METHODS**			
Protocol and registration	5	Indicate if a review protocol exists, if and where it can be accessed (e.g., Web address), and, if available, provide registration information including registration number.	3
Eligibility criteria	6	Specify study characteristics (e.g., PICOS, length of follow-up) and report characteristics (e.g., years considered, language, publication status) used as criteria for eligibility, giving rationale.	3-4
Information sources	7	Describe all information sources (e.g., databases with dates of coverage, contact with study authors to identify additional studies) in the search and date last searched.	4
Search	8	Present full electronic search strategy for at least one database, including any limits used, such that it could be repeated.	Supplementary material (SM) 4
Study selection	9	State the process for selecting studies (i.e., screening, eligibility, included in systematic review, and, if applicable, included in the meta-analysis).	3-4
Data collection process	10	Describe method of data extraction from reports (e.g., piloted forms, independently, in duplicate) and any processes for obtaining and confirming data from investigators.	3-5
Data items	11	List and define all variables for which data were sought (e.g., PICOS, funding sources) and any assumptions and simplifications made.	3-5
Risk of bias in individual studies	12	Describe methods used for assessing risk of bias of individual studies (including specification of whether this was done at the study or outcome level), and how this information is to be used in any data synthesis.	5-8; SM 6
Summary measures	13	State the principal summary measures (e.g., risk ratio, difference in means).	7-11
Synthesis of results	14	Describe the methods of handling data and combining results of studies, if done, including measures of consistency (e.g., I^2) for each meta-analysis.	Not applicable
Risk of bias across studies	15	Specify any assessment of risk of bias that may affect the cumulative evidence (e.g., publication bias, selective reporting within studies).	5-8

Additional analyses	16	Describe methods of additional analyses (e.g., sensitivity or subgroup analyses, meta-regression), if done, indicating which were pre-specified.	Not applicable
**RESULTS**			
Study selection	17	Give numbers of studies screened, assessed for eligibility, and included in the review, with reasons for exclusions at each stage, ideally with a flow diagram.	Figure 1
Study characteristics	18	For each study, present characteristics for which data were extracted (e.g., study size, PICOS, follow-up period) and provide the citations.	Tables 3; Figure 2 and SM 5a and 5b
Risk of bias within studies	19	Present data on risk of bias of each study and, if available, any outcome level assessment (see item 12).	Tables 1 & 2; SM 6
Results of individual studies	20	For all outcomes considered (benefits or harms), present, for each study: (a) simple summary data for each intervention group (b) effect estimates and confidence intervals, ideally with a forest plot.	Not applicable
Synthesis of results	21	Present results of each meta-analysis done, including confidence intervals and measures of consistency.	Not applicable
Risk of bias across studies	22	Present results of any assessment of risk of bias across studies (see Item 15).	Tables 1-2; SM 6
Additional analysis	23	Give results of additional analyses, if done (e.g., sensitivity or subgroup analyses, meta-regression [see Item 16]).	Not applicable
**DISCUSSION**			
Summary of evidence	24	Summarize the main findings including the strength of evidence for each main outcome; consider their relevance to key groups (e.g., healthcare providers, users, and policy makers).	12-14
Limitations	25	Discuss limitations at study and outcome level (e.g., risk of bias), and at review-level (e.g., incomplete retrieval of identified research, reporting bias).	14
Conclusions	26	Provide a general interpretation of the results in the context of other evidence, and implications for future research.	12-14
**FUNDING**			
Funding	27	Describe sources of funding for the systematic review and other support (e.g., supply of data); role of funders for the systematic review.	Not applicable

Table S2. PRISMA Harms checklist items [2].

Section/topic (page no)	Item	PRISMA checklist item	PRISMA harms (minimum)	Recommendations for reporting harms in systematic reviews (desirable)	Check if done
			**Title**		
Title (3)	1	Identify the report as a systematic review, meta-analysis, or both.	Specifically mention "harms" or other related terms, or the harm of interest in the review.	—	Yes<br>Page 1
			**Abstract**		
Structured summary (4)	2	Provide a structured summary including, as applicable: background; objectives; data sources; study eligibility criteria, participants, and interventions; study appraisal and synthesis methods; results; limitations; conclusions and implications of key findings; systematic review registration number.	—	Abstracts should report any analysis of harms undertaken in the review, if harms are a primary or secondary outcome.	Yes<br>Page 1
			**Introduction**		
Rationale (5)	3	Describe the rationale for the review in the context of what is already known.	—	It should clearly describe in introduction or in methods section which events are considered harms and provide a clear rationale for the specific harm(s), condition(s), and patient group(s) included in the review.	Yes<br>Page 2-3
Objectives (5)	4	Provide an explicit statement of questions being addressed with reference to participants, interventions, comparisons, outcomes, and study design (PICOS).	—	PICOS format should be specified, although in systematic reviews of harms the selection criteria for P, C, and O may be very broad (same intervention may have been used for heterogeneous indications in a diverse range of patients)	Yes<br>Page 3
			**Methods**		
Protocol and registration (6)	5	Indicate if a review protocol exists, if and where it can be accessed (eg, web address), and, if available, provide registration information including registration number.	—	No specific additional information is required for systematic reviews of harms.	Yes<br>(PROSPERO)<br>Page 3
Eligibility criteria (6)	6	Specify study characteristics (eg, PICOS, length of follow-up) and report characteristics (eg, years considered, language, publication	—	Report how handled relevant studies (based on population and intervention) when the outcomes of interest were not reported.	Yes<br>Page 3-4

		status) used as criteria for eligibility, giving rationale.		Report choices for specific study designs and length of follow-up.	
Information sources (7)	7	Describe all information sources (eg, databases with dates of coverage, contact with study authors to identify additional studies) in the search and date last searched.	—	Report if only searched for published data, or also sought data from unpublished sources, from authors, drug manufacturers and regulatory agencies. If includes unpublished data, provide the source and the process of obtaining it.	Yes<br>Page 4-5
Search (7)	8	Present full electronic search strategy for at least one database, including any limits used, such that it could be repeated.	—	If additional searches were used specifically to identify adverse events, authors should present the full search process so it can be replicated.	Yes<br>Page 4<br>Supplementary material (SM 4)
Study selection (8)	9	State the process for selecting studies (ie, screening, eligibility, included in systematic review, and, if applicable, included in the meta-analysis).	—	If only included studies reporting on adverse events of interest, defined if screening was based on adverse event reporting in title/abstract or full text. If no harms reported in the text, report if any attempt was made to retrieve relevant data from authors.	Yes<br>Page 3-5
Data collection process (9)	10	Describe method of data extraction from reports (eg, piloted forms, independently, in duplicate) and any processes for obtaining and confirming data from investigators.	—	No specific additional information is required for systematic reviews of harms.	Yes<br>Page 3-7
Data items (9)	11	List and define all variables for which data were sought (eg, PICOS, funding sources) and any assumptions and simplifications made.	—	Report the definition of the harm and seriousness used by each included study (if applicable). Report if multiple events occurred in the same individuals, if this information is available. Consider if the harm may be related to factors associated with participants (eg, age, sex, use of medications) or provider (eg, years of practice, level of training). Specify if information was extracted and how it was used in subsequent results. Specify if extracted details regarding the specific methods used to capture harms (active/passive and timing of adverse event).	Yes<br>Page 3-7
Risk of bias in individual studies (10)	12	Describe methods used for assessing risk of bias of individual studies (including specification of whether this was done at the study or outcome level), and how this information is to be used in any data synthesis.	—	The risk of bias assessment should be considered separately for outcomes of benefit and harms.	Yes<br>Table 1 & SM 6
Summary measures (11)	13	State the principal summary measures (eg, risk ratio, difference in means).	—	No specific additional information is required for systematic reviews of harms.	Yes<br>Table 3; Figure 2 & SM 8

Synthesis of results (11)	14	Describe the methods of handling data and combining results of studies, if done, including measures of consistency (eg, I^2) for each meta-analysis.	Specify how zero events were handled, if relevant.		Not applicable
Risk of bias across studies (11)	15	Specify any assessment of risk of bias that may affect the cumulative evidence (eg, publication bias, selective reporting within studies).	—	Present the extent of missing information (studies without harms outcomes), any factors that may account for their absence, and whether these reasons may be related to the results.	Yes<br>Table 1 & SM 6
Additional analyses (12)	16	Describe methods of additional analyses (eg, sensitivity or subgroup analyses, meta-regression), if done, indicating which were prespecified.	—	Sensitivity analyses may be affected by different definitions, grading, and attribution of adverse events, as adverse events are typically infrequent or reported using heterogeneous classifications. Report the number of participants and studies included in each subgroup.	Yes<br>SM 5a & 5b
			**Results**		
Study selection (13)	17	Give numbers of studies screened, assessed for eligibility, and included in the review, with reasons for exclusions at each stage, ideally with a flow diagram.	—	If a review addresses both efficacy and harms, display a flow diagram specific for each (efficacy and harm).	Yes<br>Figure 1
Study characteristics (14)	18	For each study, present characteristics for which data were extracted (eg, study size, PICOS, follow-up period) and provide the citations.	Define each harm addressed, how it was ascertained (eg, patient report, active search), and over what time period.	Add additional characteristics to: "P" (population) patient risk factors that were considered as possibly affecting the risk of the harm outcome. "I" (intervention) professional expertise/skills if relevant (for example if the intervention is a procedure). "T" (time) timing of all harm's assessments and the length of follow-up.	Yes<br>Page 3-5
Risk of bias within studies (15)	19	Present data on risk of bias of each study and, if available, any outcome level assessment (see item 12).	—	Consider the possible sources of biases that could affect the specific harm under consideration within the review. Sample selection, dropouts and measurement of adverse events should be evaluated separately from the outcomes of benefit as described in item 12, above.	Yes<br>Table 1 & SM 6
Results of individual studies (16)	20	For all outcomes considered (benefits or harms), present, for each study: (a) simple summary data for each intervention group (b) effect estimates and confidence intervals, ideally with a forest plot.	—	Report the actual numbers of adverse events in each study, separately for each intervention.	Yes<br>SM 5a and 5b
Synthesis of results (17)	21	Present results of each meta-analysis done, including confidence intervals and measures of consistency.	Describe any assessment of possible causality.	If included data from unpublished sources, report clearly the data source and the impact of these studies to the final systematic review.	Not applicable

Risk of bias across studies (18)	22	Present results of any assessment of risk of bias across studies (see item 15).	—	No specific additional information is required for systematic reviews of harms. See item 15 above.	Not applicable
Additional analysis (18)	23	Give results of additional analyses, if done (eg, sensitivity or subgroup analyses, meta-regression (see item 16)).	—	No specific additional information is required for systematic reviews of harms.	Not applicable
			**Discussion**		
Summary of evidence (18)	24	Summarise the main findings including the strength of evidence for each main outcome; consider their relevance to key groups (eg, healthcare providers, users, and policy makers).	—	No specific additional information is required for systematic reviews of harms.	Yes<br>Page 12-14
Limitations (18)	25	Discuss limitations at study and outcome level (eg, risk of bias), and at review level (eg, incomplete retrieval of identified research, reporting bias).	—	Recognise possible limitations of meta-analysis for rare adverse events (ie, quality and quantity of data), issues noted previously related to collection and reporting.	Yes<br>Page 14
Conclusions (19)	26	Provide a general interpretation of the results in the context of other evidence, and implications for future research.	—	State conclusions in coherence with the review findings. When adverse events were not identified we caution against the conclusion that the intervention is “safe,” when, in reality, its safety remains unknown.	Yes<br>Page 12-14
			**Funding**		
Funding (19)	27	Describe sources of funding for the systematic review and other support (eg, supply of data); role of funders for the systematic review.	—	No specific additional information is required for systematic reviews of harms.	Not applicable<br>Page 15

Table S3. The PRISMA for Abstracts checklist [3].

TITLE	CHECKLIST ITEM	REPORTED ON PAGE #
1. Title:	Identify the report as a systematic review, meta-analysis, or both.	1
**BACKGROUND**		
2. Objectives:	The research question including components such as participants, interventions, comparators, and outcomes.	1
**METHODS**		
3. Eligibility criteria:	Study and report characteristics used as criteria for inclusion.	1
4. Information sources:	Key databases searched and search dates.	1
5. Risk of bias:	Methods of assessing risk of bias.	1
**RESULTS**		
6. Included studies:	Number and type of included studies and participants and relevant characteristics of studies.	1
7. Synthesis of results:	Results for main outcomes (benefits and harms), preferably indicating the number of studies and participants for each. If meta-analysis was done, include summary measures and confidence intervals.	1
8. Description of the effect:	Direction of the effect (i.e. which group is favoured) and size of the effect in terms meaningful to clinicians and patients.	Not applicable
**DISCUSSION**		
9. Strengths and Limitations of evidence:	Brief summary of strengths and limitations of evidence (e.g. inconsistency, imprecision, indirectness, or risk of bias, other supporting or conflicting evidence)	1
10. Interpretation:	General interpretation of the results and important implications	1
**OTHER**		
11. Funding:	Primary source of funding for the review.	None
12. Registration:	Registration number and registry name.	PROSPERO CRD42017076736

Table S4. Search strategy.

Database/ Search strategy PubMed	Notes	Items found	Date Last Updated
("Depression"[Mesh] OR "Depressive Disorder"[Mesh] OR "Bipolar Disorder"[Mesh] OR "Adjustment Disorders"[Mesh] OR "Affective Disorders, Psychotic"[Mesh] OR Depress*[Text Word] OR Melancholi*[Text Word] OR "Involutional Psychosis"[Text Word] OR "Involutional Psychoses"[Text Word] OR "Involutional Paraphrenia"[Text Word] OR "Involutional Paraphrenias"[Text Word] OR Dysthymi*[Text Word] OR "Reactive Disorders"[Text Word] OR "Reactive Disorder"[Text Word] OR "Adjustment Disorder"[Text Word] OR "Adjustment Disorders"[Text Word] OR "Anniversary Reaction"[Text Word] OR "Anniversary Reactions"[Text Word] OR "Transient Situational Disturbance"[Text Word] OR Bipolar[Text Word] OR Manic[Text Word] OR Mania[Text Word] OR Manias[Text Word] OR "Affective Psychoses"[Text Word] OR "Affective Psychosis"[Text Word] OR "Psychotic Affective Disorders"[Text Word] OR "Psychotic Mood Disorders"[Text Word] OR "Psychotic Mood Disorder"[Text Word] OR "Psychotic Affective Disorder"[Text Word] Or "Anxiety"[Mesh] OR "Anxiety Disorders"[Mesh] OR Hypervigilance[Text Word] OR Nervousness[Text Word] OR Anxiety[Text Word] OR Anxieties[Text Word] OR " Fear*"[Text Word] OR Phobia[Text Word] OR GAD[Text Word] OR SAD[Text Word] OR PD[Text Word] OR Panic[Text Word] OR "overanxious disorder"[Text Word] OR OAD[Text Word] OR obsessive[Text Word] OR OCD[Text Word] OR obsession[Text Word] OR compulsion[Text Word] OR compulsive[Text Word] or "Suicide"[Mesh] OR "Self-Injurious Behavior"[Mesh] OR Suicid*[Text Word] OR Parasuicide[Text Word] OR Parasuicides[Text Word] OR "Deliberate self harm"[Text Word] OR "Deliberate self-harm"[Text Word] OR DSH[Text Word] OR "Self injurious behavior"[Text Word] OR "Self Injurious"[Text Word] OR "Self-Injurious"[Text Word] OR "Self Injury"[Text Word] OR "Self-Injury"[Text Word] OR "Self Injuries"[Text Word] OR "Self-Injuries"[Text Word] OR "Self-Destructive"[Text Word] OR "Self Destructive"[Text Word] OR "Non-Suicidal"[Text Word] OR "Non Suicidal"[Text Word] OR NonSuicidal[Text Word] OR Self-Harm[Text Word] OR "Self Harm"[Text Word] OR self-inflict[Text Word] OR "self inflict"[Text Word] OR selfinflict[Text Word] OR selfmutilation[Text Word] OR "self-mutilation"[Text Word] OR "self mutilation"[Text Word] OR "suicide attempt"[Text Word] OR "non suicidal"[Text Word] OR non-suicidal[Text Word] OR nonsuicidal[Text Word] OR "suicide ideation"[Text Word] OR "suicide-ideation"[Text Word) AND ("Qatar"[Mesh] OR "Bahrain"[Mesh] OR "Oman"[Mesh] OR "Saudi Arabia"[Mesh] OR "Kuwait"[Mesh] OR "United Arab Emirates"[Mesh] OR "Yemen"[Mesh] OR "Egypt"[Mesh] OR "Jordan"[Mesh] OR "Lebanon"[Mesh] OR "Syria"[Mesh] OR "Iraq"[Mesh] OR "Algeria"[Mesh] OR "Libya"[Mesh] OR "Morocco"[Mesh] OR "Tunisia"[Mesh] OR "Djibouti"[Mesh] OR "Sudan"[Mesh] OR "South Sudan"[Mesh] OR "Pakistan"[Mesh] OR "Africa, Northern"[Mesh] OR "Africa, Eastern"[Mesh] OR "middle east"[Mesh] OR "Arabs"[Mesh] OR "UAE"[Text Word] OR "U.A.E"[Text Word] OR Emirat*[Text Word] OR "United Arab Emirates"[Text Word] OR Qatar*[Text Word] OR Oman*[Text Word] OR (Saudi[Text Word] AND Arabia*[Text Word]) OR Saudi*[Text Word] OR Kuwait*[Text Word] OR Bahrain*[Text Word] OR Yemen*[Text Word] OR Egypt*[Text Word] OR Jordan*[Text Word] OR Leban*[Text Word] OR Syria*[Text Word] OR Iraq*[Text Word] OR "West Bank"[Text Word] OR Gaza*[Text Word] OR Palestin*[Text Word] OR Algeria*[Text Word] OR Libya*[Text Word] OR Morocc*[Text Word] OR Tunis*[Text Word] OR Djibouti*[Text Word] OR Sudan*[Text Word] OR South Sudan*[Text Word] OR Pakistan*[Text Word] OR (North[Text Word] AND Africa*[Text Word]) OR North-Africa*[Text Word] OR ("Africa"[Text Word] AND "Northern"[Text Word]) OR "Northern Africa"[Text Word] OR "East Africa"[Text Word] OR ("Africa"[Text Word] AND "Eastern"[Text Word]) OR "Maghreb"[Text Word] OR "Maghrib"[Text Word] OR Arab*[Text Word] OR Bedouin*[Text Word] OR	Depression + Anxiety+ Suicide, All MENA With filters: Systematic Reviews, Meta-Analysis & Reviews; From 2008	359	7/11/19

"Gulf Cooperation Council"[Text Word] OR "GCC"[Text Word] OR "Middle East"[Text Word]			
**Database/ Search strategy**<br>**Embase**	**Notes**	**Items found**	**Date Last Updated**
(exp depression/ or Depressi*.mp. or bipolar disorder?.mp. or Adjustment Disorder?.mp. or exp adjustment disorder/ or dysphoria.mp. or dysthymia.mp. or melancholia.mp. or mourning syndrome.mp. or Perry syndrome.mp. or pseudodementia.mp. or seasonal affective disorder?.mp. or affective.mp. or exp affective psychosis/ or exp bipolar disorder/ or Melancholi*.mp. or Involutional Psychosis.mp. or Involutional Psychoses.mp. or Involutional Paraphrenia?.mp. or Dysthymi*.mp. or Reactive Disorder?.mp. or Anniversary Reaction?.mp. or Transient Situational Disturbance.mp. or Manic.mp. or Mania?.mp. or Psychotic Mood Disorder?.mp. or exp anxiety disorder/ or anxiety.mp. or exp anxiety/ or Hypervigilance.mp. Or Anxiety Disorder?.mp. Or Nervousness.mp. Or jitteriness.mp. or nervositas.mp. or nervosity.mp. or neurosis.mp. or phobic.mp. or Anxieties.mp. Or Phobia?.mp. Or GAD.mp. Or SAD.mp. Or Panic.mp. Or overanxious disorder.mp. Or OCD.mp. Or obsessi*.mp. Or compulsi*.mp. or fear.mp. or exp suicidal behavior/ or Suicid*.mp. Or Deliberate self harm.mp. Or Deliberate self-harm.mp. Or Parasuicide?.mp. Or DSH.mp. Or Self injurious behavior.mp. Or Self Injurious.mp. Or Self-Injurious.mp. Or Self Injury.mp. Or Self-Injury.mp. Or Self Injuries.mp. or Self-Injuries.mp. or Self-Destructive.mp. or Self Destructive.mp. or Non-Suicidal.mp. or Non Suicidal.mp. or NonSuicidal.mp. or Self-Harm.mp. or Self Harm.mp. or artificial skin lesion.mp. or mutilation,auto.mp. or self inflicted injuries.mp. or self inflicted injury.mp. or self mutilation.mp. or self wounding.mp.) AND (exp Middle East/ or exp North Africa/ or exp Arab/ or exp Djibouti/ or exp Pakistan/ or exp Sudan/ or exp South Sudan/ or Middle East.mp. or North Africa.mp. or EMRO.mp. or Eastern Mediterranean.mp. or Arab.mp. or Arabs.mp. or Arab World.mp. or Algeria*.mp. or Bahrain*.mp. or Djibouti.mp. or Egypt*.mp. or Jordan*.mp. or Kuwait*.mp. or Leban*.mp. or Libya*.mp. or Iraq*.mp. or Morocc*.mp. or Oman*.mp. or Pakistan*.mp. or Qatar*.mp. or Saudi*.mp. or Sudan*.mp. or Syria*.mp. or Tunisia*.mp. or United Arab Emirates.mp. or Emirat*.mp. or West Bank.mp. or Ghaza*.mp. or Gaza*.mp. or Palestin*.mp. or Yemen*.mp. or UAE.mp. or KSA.mp.)	Depression + Anxiety+ Suicide, All MENA Limiters - Date Published: 2008-current<br><br>Limited to evidence-based medicine or meta-analysis or systematic review, Human Exclude medline journals	49	7/11/19
**Database/ Search Strategy**<br>**Psycinfo**	**Notes**	**Items found**	**Date Last Updated**
(DE "Arabs" OR MA qatar OR bahrain OR oman OR "Saudi arabia" OR saudi OR Kuwait OR Arabia* OR "united arab emirates" OR yemen OR egypt OR jordan OR lebanon OR syria OR Iraq OR algeria OR libya OR morocco OR tunisia OR djibouti OR sudan OR "South sudan" OR pakistan OR "North africa" OR "northern africa" OR "east africa" OR "eastern africa" OR bedouin OR maghreb OR maghrib OR "gulf cooperation council" OR GCC OR SU qatar OR bahrain OR oman OR "Saudi arabia" OR saudi OR Kuwait OR Arabia* OR "united arab emirates" OR yemen OR egypt OR jordan OR lebanon OR syria OR Iraq OR algeria OR libya OR morocco OR tunisia OR djibouti OR sudan OR "South sudan" OR pakistan OR "North africa" OR "northern africa" OR "east africa" OR "eastern africa" OR bedouin OR maghreb OR maghrib OR "gulf cooperation council" OR GCC) OR (TX UAE OR TX U.A.E OR TX Emirat* OR TX United Arab Emirates OR TX Qatar* OR TX Oman* OR TX Saudi Arabia* OR TX Saudi* OR TX Kuwait* OR TX Bahrain* OR TX Yemen* OR TX Egypt* OR TX Jordan* OR TX Leban* OR TX Syria* OR TX Iraq* OR TX West Bank OR TX Gaza* OR TX Palestin* OR TX Algeria* OR TX Libya* OR TX Morocc* OR TX Tunis* OR TX Djibouti* OR TX Sudan*	Depression, All MENA Limiters - Date Published: 20080101-20191231<br><br>Narrow by Methodology: -	330	7/8/19

OR TX South Sudan* OR TX Pakistan* OR TX North Africa* OR TX North-Africa* OR TX "Northern Africa" OR RX "East Africa" OR TX "Eastern Africa" OR TX Maghreb OR TX Maghrib OR TX Arab* OR TX Bedouin* OR TX "Gulf Cooperation Council" OR TX GCC OR TX "Middle East") OR (TX UAE OR TX U.A.E OR TX Emirat* OR TX United Arab Emirates OR TX Qatar* OR TX Oman* OR TX Saudi Arabia* OR TX Saudi* OR TX Kuwait* OR TX Bahrain* OR TX Yemen* OR TX Egypt* OR TX Jordan* OR TX Leban* OR TX Syria* OR TX Iraq* OR TX West Bank OR TX Gaza* OR TX Palestin* OR TX Algeria* OR TX Libya* OR TX Morocc* OR TX Tunis* OR TX Djibouti* OR TX Sudan* OR TX South Sudan* OR TX Pakistan* OR TX North Africa* OR TX North-Africa* OR TX "Northern Africa" OR RX "East Africa" OR TX "Eastern Africa" OR TX Maghreb OR TX Maghrib OR TX Arab* OR TX Bedouin* OR TX "Gulf Cooperation Council" OR TX GCC OR TX "Middle East") AND (DE "Major Depression" OR DE "Anaclitic Depression" OR DE "Dysthymic Disorder" OR DE "Endogenous Depression" OR DE "Late Life Depression" OR DE "Postpartum Depression" OR DE "Reactive Depression" OR DE "Recurrent Depression" OR DE "Treatment Resistant Depression") OR (DE "Depression (Emotion)")) OR (DE "Bipolar Disorder" OR DE "Bipolar I Disorder" OR DE "Bipolar II Disorder" OR DE "Cyclothymic Disorder" OR DE "Mania")) OR (DE "Mania" OR DE "Hypomania")) OR (DE "Affective Disorders" OR DE "Disruptive Mood Dysregulation Disorder" OR DE "Major Depression" OR DE "Seasonal Affective Disorder")) OR (DE "Adjustment Disorders") OR DE "Affective Disorders" OR DE "Disruptive Mood Dysregulation Disorder" OR DE "Major Depression" OR DE "Seasonal Affective Disorder" OR TX Depressi* OR Dysthymi* OR Bipolar OR Mani* OR "Seasonal affective disorder" OR Melanchol* OR "Adjustment disorder" OR coping OR cope OR cyclothymi* OR "Internalizing symptom" OR "Internalizing symptoms" OR dysphor* OR "Reactive disorder" OR "Mood dysregulation" OR "mood disorder" OR "mood disorders" OR Affective psychos?s)	meta analysis<br><br>Narrow by Methodology: - systematic review<br><br>Narrow by Methodology: - literature review<br><br>Narrow by Methodology: - metasyntheses		
**Database/ Search Strategy**<br>**Psycinfo**	**Notes**	**Items found**	**Date Last Updated**
(DE "Arabs" OR MA qatar OR bahrain OR oman OR "Saudi arabia" OR saudi OR Kuwait OR Arabia* OR "united arab emirates" OR yemen OR egypt OR jordan OR lebanon OR syria OR Iraq OR algeria OR libya OR morocco OR tunisia OR djibouti OR sudan OR "South sudan" OR pakistan OR "North africa" OR "northern africa" OR "east africa" OR "eastern africa" OR bedouin OR maghreb OR maghrib OR "gulf cooperation council" OR GCC OR SU qatar OR bahrain OR oman OR "Saudi arabia" OR saudi OR Kuwait OR Arabia* OR "united arab emirates" OR yemen OR egypt OR jordan OR lebanon OR syria OR Iraq OR algeria OR libya OR morocco OR tunisia OR djibouti OR sudan OR "South sudan" OR pakistan OR "North africa" OR "northern africa" OR "east africa" OR "eastern africa" OR bedouin OR maghreb OR maghrib OR "gulf cooperation council" OR GCC) OR (TX UAE OR TX U.A.E OR TX Emirat* OR TX United Arab Emirates OR TX Qatar* OR TX Oman* OR TX Saudi Arabia* OR TX Saudi* OR TX Kuwait* OR TX Bahrain* OR TX Yemen* OR TX Egypt* OR TX Jordan* OR TX Leban* OR TX Syria* OR TX Iraq* OR TX West Bank OR TX Gaza* OR TX Palestin* OR TX Algeria* OR TX Libya* OR TX Morocc* OR TX Tunis* OR TX Djibouti* OR TX Sudan* OR TX South Sudan* OR TX Pakistan* OR TX North Africa* OR TX North-Africa* OR TX "Northern Africa" OR RX "East Africa" OR TX "Eastern Africa" OR TX Maghreb OR TX Maghrib OR TX Arab* OR TX Bedouin* OR TX "Gulf Cooperation Council" OR TX GCC OR TX "Middle East") OR (TX UAE OR TX U.A.E OR TX Emirat* OR TX United Arab Emirates OR TX Qatar* OR TX Oman* OR TX Saudi Arabia* OR TX Saudi* OR TX Kuwait* OR TX Bahrain* OR TX Yemen* OR TX Egypt* OR TX Jordan* OR TX Leban* OR TX Syria* OR TX Iraq* OR TX West Bank OR TX Gaza* OR TX Palestin* OR TX Algeria* OR TX Libya* OR TX Morocc* OR TX Tunis* OR TX Djibouti* OR TX Sudan* OR TX South Sudan* OR TX Pakistan* OR TX North Africa* OR TX North-Africa* OR TX "Northern Africa" OR RX "East Africa" OR TX "Eastern Africa" OR TX Maghreb OR TX Maghrib OR TX Arab* OR TX Bedouin* OR TX "Gulf Cooperation Council" OR TX GCC OR TX "Middle East") AND ((((((((DE "Anxiety" OR DE "Anxiety Sensitivity" OR DE "Computer Anxiety" OR DE "Health Anxiety" OR DE	Anxiety, All MENA Limiters - Date Published: 20080101-20191231<br><br>Narrow by Methodology: - metasynthesis<br><br>Narrow by Methodology: - meta analysis<br><br>Narrow by Methodology: -	313	7/8/19

"Mathematics Anxiety" OR DE "Performance Anxiety" OR DE "Social Anxiety" OR DE "Speech Anxiety" OR DE "Test Anxiety") OR (DE "Anxiety Disorders" OR DE "Castration Anxiety" OR DE "Death Anxiety" OR DE "Generalized Anxiety Disorder" OR DE "Obsessive Compulsive Disorder" OR DE "Panic Attack" OR DE "Panic Disorder" OR DE "Phobias" OR DE "Separation Anxiety Disorder" OR DE "Trichotillomania")) OR (DE "Anxiety Sensitivity") OR TX Emotion* OR Agitat* OR Fear* OR GAD OR Guilt* OR "Internalizing symptom" OR "Internalizing symptoms" OR internaliz* OR panic OR phobi* OR shame OR angst OR worry OR apprehensi* OR Hypochondria* OR hypervigilan* OR anxiet* OR anxious* OR SAD OR PD OR OAD OR overanxi* OR OCD OR obsessive OR compulsive OR obsession OR compulsion ")	systematic review<br><br>Narrow by Methodology: - literature review		
**Database/ Search Strategy<br>Psycinfo**	**Notes**	**Items found**	**Date Last Updated**
(DE "Arabs" OR MA qatar OR bahrain OR oman OR "Saudi arabia" OR saudi OR Kuwait OR Arabia* OR "united arab emirates" OR yemen OR egypt OR jordan OR lebanon OR syria OR Iraq OR algeria OR libya OR morocco OR tunisia OR djibouti OR sudan OR "South sudan" OR pakistan OR "North africa" OR "northern africa" OR "east africa" OR "eastern africa" OR bedouin OR maghreb OR maghrib OR "gulf cooperation council" OR GCC OR SU qatar OR bahrain OR oman OR "Saudi arabia" OR saudi OR Kuwait OR Arabia* OR "united arab emirates" OR yemen OR egypt OR jordan OR lebanon OR syria OR Iraq OR algeria OR libya OR morocco OR tunisia OR djibouti OR sudan OR "South sudan" OR pakistan OR "North africa" OR "northern africa" OR "east africa" OR "eastern africa" OR bedouin OR maghreb OR maghrib OR "gulf cooperation council" OR GCC) OR (TX UAE OR TX U.A.E OR TX Emirat* OR TX United Arab Emirates OR TX Qatar* OR TX Oman* OR TX Saudi Arabia* OR TX Saudi* OR TX Kuwait* OR TX Bahrain* OR TX Yemen* OR TX Egypt* OR TX Jordan* OR TX Leban* OR TX Syria* OR TX Iraq* OR TX West Bank OR TX Gaza* OR TX Palestin* OR TX Algeria* OR TX Libya* OR TX Morocc* OR TX Tunis* OR TX Djibouti* OR TX Sudan* OR TX South Sudan* OR TX Pakistan* OR TX North Africa* OR TX North-Africa* OR TX "Northern Africa" OR RX "East Africa" OR TX "Eastern Africa" OR TX Maghreb OR TX Maghrib OR TX Arab* OR TX Bedouin* OR TX "Gulf Cooperation Council" OR TX GCC OR TX "Middle East") AND (DE "Suicide" OR DE "Attempted Suicide" OR DE "Suicidality" OR DE "Suicidology" OR DE "Self-Poisoning" OR DE "Self-Inflicted Wounds" OR DE suicide ideation DE "Self-Injurious Behavior" OR DE "Head Banging" OR DE "Self-Inflicted Wounds" OR DE "Self-Mutilation" OR DE "Self-Poisoning" OR TX Suicid* OR selfharm OR "self harm" OR self-harm OR "self-harm" OR "self injury" OR self-injury OR "self-injury" OR selfinjury OR "self injurious" OR selfinjurious OR self-injurious OR "self-injurious" OR self-mutilation OR "self-mutilation" OR selfmutilation OR "self mutilation" OR "suicide ideation" OR "Suicide-ideation" OR Suicide-ideation OR Suicideideation OR "suicidal ideation" OR "Suicidal-ideation" OR "suicidalideation" OR Suicidal-ideation OR selfinflicted OR self-inflicted OR "self inflicted" OR "self-inflicted" OR selfdestructive OR self-destructive OR "self destructive" OR "self-destructive" OR "non suicide" OR "non-suicide" OR non-suicide OR nonsuicide OR non-suicidal OR "non-suicidal" OR nonsuicidal OR "non suicidal" OR "suicide ideation" OR "suicidal ideation" OR self-inflicted OR "self-inflicted" OR "self inflicted" OR "self inflict" OR self-inflict OR "self-inflict")	Suicide, All MENA Limiters - Date Published: 20080101 - 20191231<br><br>Narrow by Methodology: - metasynthesis<br><br>Narrow by Methodology: - meta analysis<br><br>Narrow by Methodology: - systematic review<br><br>Narrow by Methodology: - literature review	59	7/9/19
**Summary Search**		**1110**	7/9/19

Table 5. Characteristics of included prevalence studies and reported prevalence of perinatal mental illness and suicides in the Middle East and North Africa Supplementary material.

Systematic Review	Primary study	Sample Size	Country	Study Population	Specific Health Outcome	Study Period	Study setting	Sampling method	Study design	Instrument used/ Suicide data source	Reported Prevalence (%)
**Prevalence of perinatal mental illness**											
Afzal and Khalid, 2016 [4]	Rasheed, 1988 [5]	103	Pakistan	PP	Postnatal blues and depression	Unclear	Unclear	Unclear	Cross sectional	Unclear	54.36%
Afzal and Khalid, 2016 [4]	Khalid, 1989 [6]	Unclear	Pakistan	PP	Postnatal blues and depression	Unclear	Unclear	Unclear	Cross sectional	Gordon's maternity blues Questionnaire (1984) Pitt's Questionnaire (1968)	Unclear
Afzal and Khalid, 2016 [4]	Sarwar, 1990 [7]	50	Pakistan	PP	Depression	Unclear	Unclear	Unclear	Longitudinal	BDI	Unclear
Afzal and Khalid, 2016 [4]	Habib, 1997 [8]	30	Pakistan	PP	Depression	Unclear	Unclear	Unclear	Cross sectional	Unclear	20%
Afzal and Khalid, 2016 [4]	Ahmed, 2005 [9]	90	Pakistan	PP	Depression	Unclear	Hospital	Convenience	Cross sectional	EPDS	27%
Afzal and Khalid, 2016 [4]	Muneer, et al., 2009 [10]	154	Pakistan	PP	Depression	Unclear	Unclear	Unclear	Cross-sectional	EPDS (Urdu version) & Hamilton rating scale for Depression	33%

Afzal and Khalid, 2016 [4] Mahendran, et al., 2019 [11]	Karmaliani, et al., 2009 [12]	1368	Pakistan	AP: 2nd trimester	Depression and Anxiety	Unclear	Health facility and Community	Non-probability	Cross-sectional	AKUADS>13	17.97%
Afzal and Khalid, 2016 [4]	Haider, 2010 [13]	213	Pakistan	AP	Depression	Unclear	Unclear	Unclear	Cross-sectional	EPDS	42.7%
Afzal and Khalid, 2016 [4]	Yasmeen, et al., 2010 [14]	100	Pakistan	PP	Depression	Unclear	Hospital	Unclear	Cross-sectional	EPDS	41%
Afzal and Khalid, 2016 [4]	Shah, et al., 2011 [15]	128	Pakistan	AP	Depression	Unclear	Unclear	Unclear	Cross-sectional	EPDS	46.8%
Alhasanat and Fry-McComish, 2015 [16]	Abou-Saleh and Ghubash, 1997 [17]	95	UAE/Dubai	PP- 7 days	Depression	Unclear	Unclear	Unclear	Cross-sectional	EPDS	18%
Alhasanat and Fry-McComish, 2015 [16] Amber Haque, 2015 [18] Fisher, et al., 2012 [19] Sawyer, et al., 2010 [20]	Agoub, et al., 2005 [22]	144	Morocco	PP: T1:2 weeks	Depression	Unclear	Primary healthcare centres	Consecutive	Longitudinal	MINI, EPDS (>12) DSM IV	T1: 18.7% (MINI) 20.1% (EPDS)
	Agoub, Moussaoui and Battas, 2005 [22]	144	Morocco	PP: T2: 6 weeks	Depression	Unclear	Primary healthcare centres	Consecutive	Longitudinal	MINI DSM IV	T2: 6.9%
	Agoub, Moussaoui and Battas, 2005 [22]	144	Morocco	PP: T3: 6 months	Depression	Unclear	Primary healthcare centres	Consecutive	Longitudinal	MINI DSM IV	T3: 11.8%

Shorey, et al., 2018 [21]	Agoub, Moussaoui and Battas, 2005 [22]	144	Morocco	PP: T4: 9 months	Depression	Unclear	Primary healthcare centres	Consecutive	Longitudinal	MINI DSM IV	T4: 5.6%
Alhasanat and Fry-McComish, 2015 [16]	Al Dallal et al., 2012	237	Bahrain	PP: 8 weeks	Depression	Unclear	Unclear	Simple random sampling	Cross-sectional	EPDS>12	37.1%
Alhasanat and Fry-McComish, 2015 [16]	Bener, et al., 2012 [23]	1379	Qatar	PP <6 months	Depression	Unclear	Unclear	Unclear	Cross-sectional	EPDS	17.6%
Alhasanat and Fry-McComish, 2015 [16] Amber Haque, 2015 [18] Klainin and Arthur, 2009 [24]	Chaaya, et al., 2002 [25]	396	Lebanon	PP T1: 24 hrs T2: 3-5 months	Depression	Unclear	Hospitals, Community	Convenience	Correlational	EPDS>=12	21%
Alhasanat and Fry-McComish, 2015 [16] Amber Haque, 2015 [18] Klainin et al., 2009 Klainin and	Green, et al., 2006 [26]	T0= 125 T1= 86 T2= 56	UAE	PP: T1: 3 months T2: 6 months	Depression	Unclear	Government maternity hospital	Convenience	Longitudinal/ Correlational	EPDS>=13 Arabic version<br>EPDS 10-12	T1: 22.0% T2: 12.5%<br>T1: 22.1% T2: 19.6%

Arthur, 2009 [24] Shorey et al., 2018 Shorey, Chee, Ng, Chan, Tam and Chong, 2018 [21]											
Alhasanat and Fry-McComish, 2015 [16] Amber Haque, 2015 [18] Shorey, Chee, Ng, Chan, Tam and Chong, 2018 [21]	Hamdan and Tamim, 2011 [27]	137	UAE	AP :2nd, 3rd trimesters PP T1: 2 months T2: 4 months	Depression	Unclear	Maternal and Child Health Centre	Convenience ; two-stage sampling	Longitudinal	Depressive symptoms-BDI Anxiety Symptoms-BAI Stressful life events Inventory Postpartum Depression -MINI (Diagnosis) -EPDS (>=10) (Screening)	10.1% (MINI) 85.6% (EPDS)
Alhasanat and Fry-McComish, 2015 [16]	Alami, et al., 2006 [28]	100	Morocco	PP	Depression	Unclear	Unclear	Unclear	Longitudinal	EPDS, MINI	21%, 19.2%
Alhasanat and Fry-McComish, 2015 [16]	Mohammad, et al., 2011 [29]	353	Jordan	AP: 3rd trimester, PP: T1: 6-8 weeks T2: 6 months	Depression		Hospital and health centres		Longitudinal	EPDS MSSS, CWS, DASS-21, PSES PKS	19% antenatal, T1: 22.1% T2: 21.2%

Amber Haque, 2015 [18]				Age (18-45)							
Alhasanat and Fry-McComish , 2015 [16]	Saleh el, et al., 2013 [30]	120	Egypt	PP	Depression	Unclear	Unclear	Unclear	Cross-sectional	EPDS	17.9%
Alhasanat and Fry-McComish , 2015 [16]	Yehia, et al., 2013 [31]	300	Jordan	PP	Depression	Unclear	Military Hospital	Unclear	Cross-sectional	EPDS	83% symptoms of Depression 39% mild symptoms 28% moderate symptoms 16% severe symptoms
Amber Haque, 2015 [18] Shorey, Chee, Ng, Chan, Tam and Chong, 2018 [21]	Al Hinai and Al Hinai, 2014 [32]	282	Oman	PP: T1: 2 weeks T2: 8 weeks	Depression	Unclear	Hospital and health center	Unclear	Cohort	EPDS>=13<br>EPDS 10-12	T1: 13.5% T2: 10.6% T1: 15.2% T2: 13.8%
Amber Haque, 2015 [18]	Abou-Saleh and Ghubash, 1997 [17]	95	UAE	PP: 2 days, 7 days	Depression	Unclear	Hospital	Unclear	Cross-sectional	SRQ EPDS	24% 18%

Amber Haque, 2015 [18]	Naglaa A. Mohamed and Maklof, 2011 [33]	110	Egypt	PP	Depression	Unclear	Hospital	Unclear	Cross-sectional	PDPI, EPDS	51.8%
Amber Haque, 2015 [18]	Bener, Gerber and Sheikh, 2012 [23]	2091	Qatar	PP: 1 year, 4 months	Depression	Unclear	Primary health care centres	Unclear	Cross-sectional	Interview DASS-21	18.60%
Amber Haque, 2015 [18]	Balaha, et al., 2009 [34]	800	Saudi Arabia	PP: 6 months	Depression	Unclear	Hospital and Primary healthcare centers	Unclear	Case- control	MINI-V	Young mother 10.1% Older mother 10.3%
Amber Haque, 2015 [18]	Masmoudi, et al., 2008 [35]	213	Tunisia	T1: first week T2: 6-10 weeks	Depression	Unclear	Hospital	Unclear	Longitudinal	EPDS	T1:19.2% T2:13.2%
Fisher, Cabral de Mello, Patel, Rahman, Tran, Holton and Holmes, 2012 [19]	Karmaliani, et al., 2006 [36]	1000	Pakistan	Pregnant women (Civil Hospital) 1000 first recruited women 20-26 weeks pregnant and living locally, identified during routine household visits of 1368/1879	Depression and anxiety	Unclear	Community	Unclear	Cross-sectional survey	AKUADS>31.5 How I feel Scale>83.5	11.5% 13.5%

				in larger study							
Fisher, Cabral de Mello, Patel, Rahman, Tran, Holton and Holmes, 2012 [19] Jones and Coast, 2013 [37] Shorey, Chee, Ng, Chan, Tam and Chong, 2018 [21] Zahidie and Jamali, 2013 [38]	Rahman, et al., 2003 [39]	632/701	Pakistan	AP: 3rd trimester	Depression	Unclear	Rural Community	Consecutive	Two stage cross-sectional survey (Prospective cohort)	Postpartum Depression-SCAN (ICD-10) PIQ LEDS BDQ	25% (6 weeks antepartum)
Fisher, Cabral de Mello, Patel, Rahman, Tran, Holton and Holmes, 2012 [19] Shorey, Chee, Ng, Chan, Tam and	Rahman, Iqbal and Harrington, 2003 [39]	541/632	Pakistan	PP: 10-12 weeks	Depression	Unclear	Rural Community	Consecutive	Two stage cross-sectional survey (Prospective cohort)	Postpartum Depression-SCAN (ICD-10) LEDS PIQ BDQ	28%

Chong, 2018 [21] Zahidie and Jamali, 2013 [38]											
Fisher, Cabral de Mello, Patel, Rahman, Tran, Holton and Holmes, 2012 [19] Jones and Coast, 2013 [37] Klainin and Arthur, 2009 [24] Shorey, Chee, Ng, Chan, Tam and Chong, 2018 [21] Zahidie and Jamali, 2013 [38]	Husain, et al., 2006 [40]	149/175 T1:175 (antena tal) T2: 149	Pakistan	PP: 12week	Depression	Unclear	Rural community	Consecutive	Cross-sectional population-based survey	EPDS>=12 Social Support- MSPSS Mental Distress- SRQ-20	T2: 36% (3 months PP)
Jones and Coast, 2013 [37]	Rahman and Creed, 2007 [41]	T1: 701 (antena tal) T2: 632 T3: 160	Pakistan	Followed up women diagnosed with prenatal	Depression	Unclear	Community	Convenience	Longitudinal	Clinical assessment in neuropsychiat ry (ICD-10 diagnosis)	T2: 94% (3 months) T3: 76% (6 months)

Klainin and Arthur, 2009 [24]<br><br>Shorey, Chee, Ng, Chan, Tam and Chong, 2018 [21]		T4: 129		depression to one year postpartum. Dependent variable was persistence of depression at 3, 6, 12 months postpartum. 56.6% followed upto 12 months had persistent depression.							T4: 62% (12 months) 57% All time points
Klainin and Arthur, 2009 [24]	Kalyani, et al., 2001 [42]	T1: 120<br>T2: 76	Pakistan	PP: T1: 2 weeks<br>T2: 2-4 weeks	Depression	Unclear	Home visit, Community	Convenience	Prospective comparative	EPDS >10	63.3%
Mahendran, Puthussery and Amalan, 2019 [11]	Ali, et al., 2012 [43]	167	Pakistan	AP: 1st,2nd, 3rd trimesters	Depression	Unclear	Health facility	Non-probability sampling	Cross-sectional	HADS score >8	16.77%
Mahendran, Puthussery and Amalan, 2019 [11]	Din, et al., 2016 [44]	230	Pakistan	AP: 3rd trimester	Depression	Unclear	Health facility	Non-probability sampling	Cross-sectional	DASS-42>9	29.13%

Mahendran, Puthussery and Amalan, 2019 [11]	Fareeha Hamid, 2008 [45]	100	Pakistan	AP: 1st,2nd, 3rd trimesters	Depression	Unclear	Health facility	Non-probability sampling	Cross-sectional	HADS score	18%
Mahendran, Puthussery and Amalan, 2019 [11]	Humayun, et al., 2013 [46]	506	Pakistan	AP: 3rd trimester	Depression	Unclear	Health Facility	Non-probability sampling	Cross-sectional	(EPDS) score>10	75.10%
Mahendran, Puthussery and Amalan, 2019 [11]	Husain, et al., 2011 [47]	1357	Pakistan	AP: 3rd trimester	Depression	Unclear	Antenatal clinics of Chiniot maternity and childcare centre	Non-probability sampling	Cohort study	EPDS>12	25.79%
Zahidie and Jamali, 2013 [38]	Husain, Parveen, Husain, Saeed, Jafri, Rahman, Tomenson and Chaudhry, 2011 [47]	149	Pakistan	PP: 12 weeks	Depression	Unclear	Maternity and childcare center	Unclear	Cohort study	EPDS	38.3%
Mahendran, Puthussery and Amalan, 2019 [11]	Haider, 2010 [13]	213	Pakistan	AP: 3rd trimester	Depression	Unclear	Health Facility	Non-probability sampling	Prospective cohort	EPDS>12	42.72%

Mahendran, Puthussery and Amalan, 2019 [11]	Sharifa Mir, 2012 [48]	328	Pakistan	AP: 1st,2nd, 3rd trimesters	Depression	Unclear	Health Facility	Non-probability sampling	Cross-sectional	AKUADS score >13	33.84%
Mahendran, Puthussery and Amalan, 2019 [11]	Syeda Rabia, 2017 [49]	520	Pakistan	AP: 1st,2nd, 3rd trimesters	Depression	Unclear	Health Facility	Non-probability sampling	Cross-sectional	HADS score >8	23.08%
Mahendran, Puthussery and Amalan, 2019 [11]	Sadaf, 2011 [50]	150	Pakistan	AP: 1st,2nd, 3rd trimesters	Depression	Unclear	Health Facility	Non-probability sampling	Cohort	HAM-D	10%
Mahendran, Puthussery and Amalan, 2019 [11]	Saeed, et al., 2016 [51]	82	Pakistan	AP: 1st,2nd, 3rd trimesters	Depression	Unclear	Health Facility	Non-probability sampling	Cohort	EPDS>9	42.68%
Mahendran, Puthussery and Amalan, 2019 [11]	Shah, Bowen, Afridi, Nowshad and Muhajarine, 2011 [15]	128	Pakistan	AP: 1st,2nd, 3rd trimesters	Depression	Unclear	Health Facility	Stratified Random sampling	Cross-sectional	EPDS>13	46.88%
Mahendran, Puthussery and	Waqas, et al., 2015 [52]	500	Pakistan	A P: 1st,2nd, 3rd trimesters	Depression	Unclear	Health Facility	Non-probability sampling	Cross-sectional	HADS>11	31.8%

Amalan, 2019 [11]										
Mahendran, Puthussery and Amalan, 2019 [11] Zahidie and Jamali, 2013 [38]	Zahidie, et al., 2011 [53]	375	Pakistan	AP: 2nd trimester	Depression	Unclear	Health Facility and community	Non-probability sampling	Mixed method study. Qualitative phase I: indepth interviews Quantitative phase II: cross-sectional survey	CES-D>16	61.07%
Sawyer, Ayers and Smith, 2010 [20]	Alami, Kadri and Berrada, 2006 [28]	100	Morocco	AP:1st trimester AP:2nd trimester AP:3rd trimester	Depression	Unclear	Unclear	Unclear	Longitudinal	MINI (DSM-IV)	17.4% 16% 15.7%
Sawyer, Ayers and Smith, 2010 [20]	Alami, Kadri and Berrada, 2006 [28]	100	Morocco	PP: 2-3 weeks 12 weeks 24 weeks 36 weeks	Depression	Unclear	Unclear	Unclear	Longitudinal	MINI (DSM-IV)	16.8% 14% 12% 6%
Shorey, Chee, Ng, Chan, Tam and Chong, 2018 [21]	Khalifa, et al., 2015 [54]	238	Sudan	PP: 3 months	Depression	Unclear	Community	Convenience	Cross-sectional population-based survey	EPDS (>=12) Arabic version MINI	9.2% (EPDS; n=238) 45% (MINI; n=40)
Stewart, 2007 [55]	Rahman, et al., 2004 [56]	265 Case-129 Control - 136	Pakistan	AP: 3rd trimester	Depression	Unclear	Rural community based	Unclear	Cohort	SCAN	25%

Stewart, 2007 [55]	Rahman, et al., 2004 [57]	172 Case-83 Control -89	Pakistan	PP	Depression	Unclear	Urban Immunisati on Clinic	Unclear	Case-control	Self-reporting questionnaire (cut-off 11/12)	41% 57% cases 25% controls
Zahidie and Jamali, 2013 [38]	Ali, et al., 2009 [58]	Unclear	Pakistan	PP: 1,2,6 and 12 months	Depression	Unclear	Urban communitie s of Karachi	Unclear	Longitudinal	AKUADS, Unclear DSM IV	28.8%
Zahidie and Jamali, 2013 [38]	Asad, et al., 2010 [59]	1369	Pakistan	AP	Depression/ Anxiety	Unclear	Primary care health centres	Unclear	Cross sectional study	AKUADS	18%
Zahidie and Jamali, 2013 [38]	Asad, Karmaliani, Sullaiman, Bann, McClure, Pasha, Wright and Goldenberg, 2010 [59]	1369	Pakistan	AP	Considered Suicide	Unclear	Primary care health centres	Unclear	Cross sectional study	AKUADS	Prevalence - 11%
Zahidie and Jamali, 2013 [38]	Asad, Karmaliani, Sullaiman, Bann, McClure, Pasha, Wright and Goldenberg, 2010 [59]	148	Pakistan	AP	Attempted Suicide	Unclear	Primary care health centres	Unclear	Cross sectional study	AKUADS	Prevalence - 45%
Additional primary studies identified	Gulamani, et al., 2013 [60]	214 34 preter m mother s	Pakistan	PP: 4-6 weeks	Depression	1 May 2010 to 30 June 2010	Unclear	Consecutive	Cohort study	EPDS PBQ PSS	35.3% (Preterm infants) 15.3% (Full-term infants)

		170 full-term mothers									
	Husain, et al., 2014 [61]	1357	Pakistan	AP: 3rd trimester	Depression	March 2004 to April 2005	Maternity home and childcare center	Consecutive	Cohort	PIQ EPDS- Urdu version SRQ-20	EPDS >=12 (13.4%) SRQ score of 7 (28.5%)
	Kazi, et al., 2006 [62]	292	Pakistan	AP: 1st, 2nd and 3rd trimester	Depression	Phase 1: July 2003 to May 2004 Phase 2: June 2004 to September 2004	Hospital	Unclear	Unclear	CES-D cut off >=16	39.4%
	Al-Modayfer, et al., 2015 [63]	571	Saudi Arabia	PP: 5 weeks after delivery	Depression	Unclear	Community	Unclear	Unclear	EPDS	14%
**Suicide data**											
Fuhr, et al., 2014 [64]	Farhat, et al., 2012 [65]	469	Tunisia	15-49years; Maternal deaths population	Proportion of pregnancy-related deaths/maternal deaths attributable to suicide	1999-2007	Hospitals, community	Unclear	Population-based	Modified RAMOS [Reproductive Age Mortality Study] (private and public hospital data	Proportion of pregnancy-related deaths/ maternal deaths attributabl

										only) to identify deaths. Private hospital data refers to the year 2006 only. Ascertainment of cause of death assignation: Review of hospital records and CEMD (Confidential Enquiries into Maternal Deaths) questionnaires by regional advisory board (composed of specialists in obstetrics and a national committee on maternal	e to suicides 0.64
Fuhr, Calvert, Ronsmans, Chandra, Sikander, De Silva	Amarin, et al., 2010 [66]	112	Jordan	15-49 years; Pregnancy related deaths	Proportion of pregnancy-related deaths/maternal deaths attributable to	2007-2008	Hospitals, Community	Unclear	Population-based	Reproductive Age Mortality Study (data from the Ministry of Health, civil	Proportion of pregnancy related deaths/ maternal

and Patel, 2014 [64]					domestic violence and suicide					registry, police units, forensic departments, records from the United Nations Relief and Works Agency) Cause of death: Relatives of women who died were contacted by phone to obtain information on the cause of death	deaths attributable to suicides 6.25
Fuhr, Calvert, Ronsmans, Chandra, Sikander, De Silva and Patel, 2014 [64]	Jafarey, et al., 2009 [67]	128	Pakistan	15-49 years; "Pregnancy-related deaths (retrospective) and pregnant women (prospective)"	Proportion of pregnancy-related deaths/maternal deaths attributable to domestic violence and suicide	2005-2007	Hospitals, community	Unclear	Population-based	Deaths were identified through monthly reports of lady health workers, health management information systems, records of hospitals, graveyards, and union information	Proportion of pregnancy-related deaths/ maternal deaths attributable to suicides 3.12

										systems, records of hospitals, graveyards, and union councils in addition to a survey with complete population coverage<br><br>Cause of deaths: Verbal autopsy (reviewed by three physicians and the principal investigator of study in case of disagreement) , consensus panel disagreement) , consensus panel	
Fuhr, Calvert, Ronsmans, Chandra, Sikander, De Silva	Ministry of Health and Population, Egypt 2001 Population, 2001 [68]	634	Egypt	15-49 years; Pregnancy related deaths	Proportion of pregnancy-related deaths/maternal deaths	2000	Hospitals, Community	Unclear	Population based	Vital routine registration data from a selection of 149 health	Proportion of pregnancy-related deaths/ maternal

and Patel, 2014 [64]	attributable to suicide	bureaus in all governorates. Deaths were identified by using data from a screening questionnaire at the health bureaus, medical records, drug prescriptions, records from hospitals and private clinics.<br><br>Cause of deaths: Verbal autopsy (cause of death data reviewed by a local advisory group), consensus panel	deaths attributabl e to suicide 5.84

Edinburgh Postnatal Depression Scale (EPDS); Schedules for Assessment in Neuropsychiatry (SCAN); Aga Khan University Anxiety Depression Scale (AKUADS); The Centre for Epidemiology Studies-Depression (CES-D) scale; Hospital anxiety Depression Scale (HADS); Personal Information Questionnaire (PIQ); Life events and difficulties Schedule (LEDS)Brief disability Questionnaire (BDQ); Multidimensional Scale of Perceived Social Support (MSPSS); Parental Bonding questionnaire (PBQ); Parental Stress Scale (PSS) Mini- International Neuropsychiatric Interview (MINI) . *cut off scores provided when available from the Systematic reviews AP- Antepartum PP- Postpartum T- Time frame

Table S6. Characteristics of studies reporting risk factors of perinatal mental health illness in the Middle East and North Africa

Systematic Review Citation	Included Study Citation	*Sample Size*	Country	Study Popl	Specific Health Outcome	Study Period	Study setting	Sampling method	Study design	Instrument used/ data source	Risk factors
Afzal and Khalid, 2016 [4]	Afzal and Khalid, 2014 [69]	*106*	Pakistan	PP	Depression	Unclear	Unclear	Unclear	Correlational	DSS<br>Social support<br>demographic<br>Performance	Social support has significant negative relationship
Afzal and Khalid, 2016 [4]	Gul, et al., 2013 [70]	*500*	Pakistan	PP	Depression	Unclear	Unclear	Unclear	Cohort study	Demographic questionnaire<br>SSDS<br>EPDS<br>DSM-IV-TR	Female gender of the newborn in the fourth pregnancy
Afzal and Khalid, 2016 [4]	Haider, 2010 [13]	*213*	Pakistan	AP, PP	Depression	Unclear	Unclear	Unclear	Cross sectional	EPDS	Marital Issues<br>Conflict with parents and parents-in-law<br>History of domestic violence<br>Psychiatric history<br>Obstetric risk factors including previous miscarriages, problematic pregnancies<br>Mothers with antenatal depression had more complicated deliveries and babies with lower birthweight and APGAR scores
Afzal and Khalid, 2016 [4]	Kalyani, Saeed, Rehman and Mubbashar, 2001 [42]	*120*	Pakistan	PP	Perinatal depression	Unclear	Unclear	Unclear	Cross sectional	Unclear	Unwanted pregnancy<br>Parity (Premiparae)<br>Living in extended family<br>Interpersonal marital difficulties<br>Early loss of mother<br>Lower level of education found to have association with PND
Afzal and Khalid, 2016 [4]	Karmaliani, Asad, Bann, Moss,	*1368*	Pakistan	AP, PP	Depression and Anxiety	Unclear	Unclear	Unclear	Cross sectional	AKUADS	Husband's unemployment (p= 0.032)

	McClure, Pasha, Wright and Goldenberg, 2009 [12]										Poor socioeconomic status (p= 0.027)<br>Education<br>First child (p= 0.002)<br>Unwanted pregnancy (p< 0.001)<br>Abuse (physical, verbal and sexual- strongest association)<br>42% history of physical and sexual abuse; 23% history of verbal abuse
Afzal and Khalid, 2016 [4]	Munaf, 2006 [71]	*50*	Pakistan	PP	Depression	Unclear	Unclear	Unclear	Cross sectional	BDI	Nuclear family
Afzal and Khalid, 2016 [4]	Muneer, Minhas, Tamiz-ud-Din Nizami, Mujeeb and Usmani, 2009 [10]	*154*	Pakistan	PP	Depression	Unclear	Unclear	Unclear	Descriptive and cross sectional	EPDS (Urdu version)<br>Hamilton rating scale for Depression	Lower Socioeconomic status<br>Low levels of education<br>Small families<br>Married with three kids<br>Married less than five years<br>Living in extended families
Afzal and Khalid, 2016 [4]	Ghazala Sadiq, 2015 [72]	*380*	Pakistan	Elderly Professional women, PP	Depression	Unclear	Unclear	Unclear	Observational, case-control and Retrospective	EPDS	Elderly and working women<br>Joint family system has negative relationship
Afzal and Khalid, 2016 [4]	Sarwar, 1990 [7]	*50*	Pakistan	PP, AP	Depression	Unclear	Unclear	Unclear	Longitudinal	BDI	Prenatal depression was a significant risk factor for postnatal depression
Afzal and Khalid, 2016 [4]	Shah, Bowen, Afridi, Nowshad and Muhajarine, 2011 [15]	*354*	Pakistan	AP	Depression	Unclear	Unclear	Unclear	Cross sectional	EPDS	Physical abuse

Afzal and Khalid, 2016 [4]	Yasmeen, Tayyaba, Chatan, Naeem, Numan and Adnan Maqsood, 2010 [14]	*100*	Pakistan	PP	Depression	Unclear	Hospitals	Unclear	Descriptive and Cross-sectional	EPDS	Previous PND<br>Marital dissatisfaction<br>Joint family system<br>Physical abuse<br>Poor socioeconomic status<br>Non-working status
Alhasanat and Fry-McComish, 2015 [16]	Abou-Saleh and Ghubash, 1997 [17]	*95*	UAE/ Dubai	PP: 7 days	Depression	Unclear	Unclear	Unclear	Cross-sectional	EPDS	Marital problems<br>Lack of assistance from husband<br>Stressful life events
Alhasanat and Fry-McComish, 2015 [16]	Agoub, Moussaoui and Battas, 2005 [22]	*144*	Morocco	PP: 2 and 6 weeks, 6 and 9 months	Depression	Unclear	Unclear	Unclear	Longitudinal	DSM IV	Poor marital relationship and lack of partner support<br>Intimate partner violence<br>Pregnancy complication<br>Stressful life events during pregnancy<br>Baby's health problems
Alhasanat and Fry-McComish, 2015 [16]	Al Dallal and Grant, 2012 [73]	*237*	Bahraini	PP	Depression	Unclear	Unclear	Simple random sampling	Cross-sectional	EPDS>12	History of depressive symptoms or antenatal depression<br>Perceived lack of partner support
Alhasanat and Fry-McComish, 2015 [16]	Bener, et al., 2012 [74]	*1379*	Qatar	PP <6 months	Depression	Unclear	Unclear	Unclear	Prospective cross-sectional study	EPDS	Age older than 35<br>Low education<br>Housewives<br>Low income
Alhasanat and Fry-McComish, 2015 [16]	Chaaya, Campbell, El Kak, Shaar, Harb and Kaddour, 2002 [25]	*396*	Lebanon	PP T1: 24 hrs T2: 3-5 months	Depression	Unclear	Unclear	Unclear	Longitudinal	EPDS>=12	Lack of Social support<br>Antenatal depression<br>Stressful life events<br>Lower educational level
Alhasanat and Fry-	Green, Broome and	*T0= 125*<br>*T1= 86*<br>*T2= 56*	UAE	PP: T1: 3 months	Depression	Unclear	Unclear	Unclear	Longitudinal,	EPDS	Non-breastfeeding<br>First child<br>Poor body image

McComish, 2015 [16]	Mirabella, 2006 [26]			T2: 6 months							Poor relationship with mother in law<br>Older age of marriage
Alhasanat and Fry-McComish, 2015 [16]	Hamdan and Tamim, 2011 [27]	*137*	UAE	PP	Depression	Unclear	Unclear	Unclear	Prospective longitudinal	EPDS	Antenatal depression (depression during both second and third trimesters)<br>Number of children<br>Religion<br>Use of formula and lack of breast feeding<br>Education of mother<br>Stressful life events
Alhasanat and Fry-McComish, 2015 [16]	Alami, Kadri and Berrada, 2006 [28]	*100*	Morocco	PP	Depression	Unclear	Unclear	Unclear	Longitudinal	EPDS<br>MINI	Baby's health problems<br>Stressful life events<br>Marital problems
Alhasanat and Fry-McComish, 2015 [16]	Mohammad, Gamble and Creedy, 2011 [29]	*353*	Jordan	AP: 3rd trimester,<br>PP: T1: 6-8 weeks<br>T2: 6 months<br>Age (18-45) years	Depression	Unclear	Unclear	Unclear	Prospective	EPDS	Antenatal depression<br>Difficult relationship with mother in law<br>Stress<br>Social support<br>Perceived low parenting knowledge (self-efficacy)
Alhasanat and Fry-McComish, 2015 [16]	Saleh el, El-Bahei, Del El-Hadidy and Zayed, 2013 [30]	*120*	Egypt	PP	Depression	Unclear	Unclear	Unclear	Comparative descriptive study	EPDS	Social support<br>Socioeconomic status<br>Non-breast feeding<br>Prior psychiatric problems
Alhasanat and Fry-McComish, 2015 [16]	Yehia, Callister and Hamdan-Mansour, 2013 [31]	*300*	Jordan	PP	Depression	Unclear	Military Hospital	Unclear	Cross-sectional	EPDS	Income<br>Intent to become pregnant<br>Birth mode<br>Family social support

Amber Haque, 2015 [18]	Abou-Saleh and Ghubash, 1997 [17]	*95*	UAE	PP: 2 days, 7 days	Depression	Unclear	Hospital	Unclear	Cross-sectional	SRQ<br>EPDS	Previous psychiatric problems<br>Marital difficulties<br>Stressful life events<br>Lack of emotional support<br>Polygamy<br>Death of one's father before the age of 13<br>Having a relative with an alcohol problem
Amber Haque, 2015 [18]	Al Hinai and Al Hinai, 2014 [32]	*282*	Oman	PP: 2, 8 weeks	Depression	Unclear	Hospital and health center	Unclear	Prospective cohort	EPDS	Younger age<br>Conflict with family members<br>Sickness of family member<br>Work difficulties
Amber Haque, 2015 [18]	Chaaya, Campbell, El Kak, Shaar, Harb and Kaddour, 2002 [25]	*396*	Lebanon	PP: 24 hrs and 3-5 months	Depression	Unclear	Hospitals	Unclear	Longitudinal	EPDS	Prenatal depression<br>Lack of social support<br>Stressful life events<br>Lifetime depression<br>Vaginal delivery<br>Little education<br>Unemployment<br>Chronic health problems
Amber Haque, 2015 [18]	Naglaa A. Mohamed and Maklof, 2011 [33]	*110*	Egypt	PP	Depression	Unclear	Hospital	Unclear	Cross-sectional	PDPI<br>EPDS	Low level of socioeconomic status<br>Feelings of depression and anxiety during pregnancy<br>History of depression<br>Lack of social support<br>Low self-esteem<br>Stressful life events
Amber Haque, 2015 [18]	Bener, Gerber and Sheikh, 2012 [23]	*2091*	Qatar	PP: 1 year, 4 months	Depression	Unclear	Primary health care centres	Unclear	Cross-sectional	Interview<br>DASS-21	Under 30 years old<br>Higher education level<br>Lower household income<br>Unplanned pregnancy<br>Lack of family support<br>Mothers as housewives

											Poor relationship with the mother-in-law
Amber Haque, 2015 [18]	Balaha, Amr, El-Gilany and Sheikh, 2009 [34]	*800*	Saudi Arabia	PP: 6 months	Depression	Unclear	Hospital and Primary healthcare centers	Unclear	Case-control	MINI-V	Comorbid psychiatric disorders in 15% of cases
Amber Haque, 2015 [18]	Green, Broome and Mirabella, 2006 [26]	*125*	UAE	PP: 3, 6 months	Depression	Unclear	Government maternity hospital	Unclear	Longitudinal,	EPDS	Not breast feeding<br>Giving birth to the first child<br>Poor self-body image and view of weight<br>Poor relationship with mother-in-law<br>Older age at marriage
Amber Haque, 2015 [18]	Hamdan and Tamim, 2011 [27]	*137*	UAE	PP: 2, 4 months	Depression	Unclear	Maternal and child health centre	Unclear	Prospective longitudinal	EPDS<br>MINI	Depression during pregnancy in both second and third trimesters<br>Number of children<br>Religion<br>Not breast feeding
Amber Haque, 2015 [18]	Masmoudi, Tabelsi, Charfeddine, Ben Ayed, Guermazzi and Jaoua, 2008 [35]	*213*	Tunisia	T1: first week<br>T2: 6-10 weeks	Depression	Unclear	Hospital	Unclear	Longitudinal	EPDS	High rate of PPD in the first stage would be contaminated by an intense postpartum blues
Amber Haque, 2015 [18]	Mohammad, Gamble and Creedy, 2011 [29]	*353*	Jordan	AP: 3rd trimester,<br>PP: 6-8 weeks, 6 months<br>Age (18-45)	Depression	Unclear	Hospital and health centres	Unclear	Cross sectional	EPDS<br>MSSS<br>CWS<br>DASS-21<br>PSES<br>PKS	Stress<br>Anxiety<br>Financial problems<br>Perceived lack of parenting skills<br>Difficult relationship with the mother-in-law<br>Unplanned pregnancy<br>Low self-efficacy
Fisher, Cabral de	Agoub, Moussaoui	*144*	Morocco	PP: 9 months	Depression	Unclear	Primary maternity	consecutive	Prospective cohort	MINI	Socioeconomic disadvantage

Mello, Patel, Rahman, Tran, Holton and Holmes, 2012 [19]	and Battas, 2005 [22]						healthcare centres				Min OR (95% CI) 2.1 (95% CI: 1.3-5.2)<br>Max OR (95% CI) 13.2 (95% CI: 5.2-33.5)<br>An unemployed partner<br>Infant is ill<br>Min OR (95% CI) 1.1 (95% CI: 0.6-2.3)<br>Max OR (95% CI) 4.5 (95% CI: 3.2-6.4)
Fisher, Cabral de Mello, Patel, Rahman, Tran, Holton and Holmes, 2012 [19]	Husain, Bevc, Husain, Chaudhry, Atif and Rahman, 2006 [40]	*149*	Pakistan	PP: 12week	Depression	Unclear	Rural communit y based	Consecutiv ely	Cross-sectional populatio n-based survey	EPDS>=11	Quality of relationship with intimate partner<br>Difficulties in intimate partner relationship<br>Min OR (95% CI) 1.96 (95% CI: 1.0-3.9)<br>Max OR (95% CI) 9.44 (95% CI: 2.4-37.8)<br>Physical violence<br>Min OR (95% CI) 2.11 (95% CI: 1.1-4.0)<br>Max OR (95% CI) 6.75 (95% CI: 2.1-2.0)
Fisher, Cabral de Mello, Patel, Rahman, Tran, Holton and Holmes, 2012 [19]	Karmaliani, Bann, Mahmood, Harris, Akhtar, Goldenberg and Moss, 2006 [36]	*1000*	Pakistan	Pregnant women (Civil Hospital) 1000 first recruited women 20-26 weeks pregnant and living locally,	Depression and Anxiety	Unclear	Civil hospital	Unclear	Cross-sectional survey	AKUADS>13.5<br>How I feel Scale>83.5	Unclear

				identified during routine household visits of 1368/1879 in larger study							
Fisher, Cabral de Mello, Patel, Rahman, Tran, Holton and Holmes, 2012 [19]	Rahman, Iqbal and Harrington, 2003 [39]	*632*	Pakistan	AP: 3rd trimester	Depression	Unclear	Rural Community	Prospective cohort	Two stage cross-sectional survey (Prospective cohort)	Postpartum Depression-SCAN (ICD-10)<br>PIQ<br>LEDS<br>BDQ	Social and economic circumstances<br>Socioeconomic disadvantage<br>Min OR (95% CI) 2.1 (95% CI: 1.3-5.2)<br>Max OR (95% CI) 13.2 (95% CI: 5.2-33.5)<br>Quality of relationship with intimate partner<br>Difficulties in intimate partner relationship including:<br>Min OR (95% CI) 1.96 (95% CI: 1.0-3.9)<br>Max OR (95% CI) 9.44 (95% CI: 2.4-37.8)<br>A partner who rejected paternity, was unsupportive, uninvolved, critical and quarrelsome or used alcohol to excess<br>Family and social relationships<br>No help from, feared or argued with in-laws<br>Min OR (95% CI) 2.14 (95% CI:1.1-4.3)<br>Max OR (95% CI) 4.4 (95% CI: 1.8-10.8)<br>Insufficient social support living in a nuclear family

											Min OR (95% CI) 2.10 (95% CI: 1.2-3.8)<br>Max OR (95% CI) 4.3 (95% CI: 1.4-13.3)<br>Having at least three children<br>Min OR (95% CI) 2.6 (95% CI: 1.1-6.3)<br>Max OR (95% CI) 4.1 (95% CI: 0.9-19.0)<br>Infant characteristics<br>Not having a child or the desired sex<br>Min OR (95% CI) 1.8 (95% CI: 1.4- 2.3)<br>Max OR (95% CI) 2.6 (95% CI: 1.2-6.5)
James-Hawkins, et al., 2019 [75]	Abdelhai and Mosleh, 2015 [76]	*376*	Egypt	AP: 1st, 2nd, 3rd trimester (1st,2nd,3 rd trimester)	Prenatal depression and anxiety	Unclear	Clinic	Systematic sampling (probability sample)	Unclear	HADS Perceived financial distress on a 5-point Likert scale (ref= no perceived financial distress)	Experiencing anxiety and depression vs. neither<br>No association between financial distress & depression / anxiety (Multivariate, logistic regression); Ua OR = 1.59, p=0.15
James-Hawkins, Shaltout, Nur, Nasrallah, Qutteina, Abdul Rahim, Hennink and Yount, 2019 [75]	Abuidhail and Abujilban, 2014 [77]	*218*	Jordan	P: 3rd trimester	Depression	Unclear	Clinic	Convenience sampling (non-probability sample)	Unclear	EPDS	Low vs high education (ref)<br>Positive association between education and prenatal depression (bivariate, means comparison); t=5.10, p=0.00

James-Hawkins, Shaltout, Nur, Nasrallah, Qutteina, Abdul Rahim, Hennink and Yount, 2019 [75]	Abujilban, et al., 2014 [78]	*218*	Jordan	AP: Literate women in third trimester	Depression	Unclear	Clinic	Convenience sampling (non-probability sample)	Observational Cross sectional	EPDS	Elementary to MA<br>Negative association between education and prenatal depression (Multivariate, regression); B=-2.2, p<0.05
James-Hawkins, Shaltout, Nur, Nasrallah, Qutteina, Abdul Rahim, Hennink and Yount, 2019 [75]	Agoub, Moussaoui and Battas, 2005 [22]	*144*	Morocco	PP: 2 and 6 weeks, 6 and 9 months	Depression	Unclear	Primary healthcare centres	consecutive	Longitudinal	MINI case vs. not	Literate vs. illiterate (ref)<br>No association between education and postnatal depression (Bivariate, means comparison); p=0.83<br>Working vs not (ref)<br>No association between employment and postnatal depression (Bivariate, means comparison); p=1.0
James-Hawkins, Shaltout, Nur, Nasrallah, Qutteina, Abdul Rahim, Hennink and Yount, 2019 [75]	Al-Azri, et al., 2016 [79]	*959*	Oman	AP: 1st,2nd,3rd trimesters	Depression	Unclear	Clinic based	Systematic sampling (probability sample)	Unclear	EPDS	Secondary or less vs. university (ref)<br>No association between education and prenatal depression (Bivariate, chi square); p=0.13<br>Housewife vs. employed (ref)<br>No association between employment and prenatal depression (Bivariate, chi square); p=0.39<br>< 500 vs. 500–1000 vs. >1000 Omani Riyal

											No association between financial resource and prenatal depression (Bivariate, chi square); p=0.08
James-Hawkins, Shaltout, Nur, Nasrallah, Qutteina, Abdul Rahim, Hennink and Yount, 2019 [75]	Al Dallal and Grant, 2012 [73]	*237*	Bahrain	PP: 8 weeks	Depression	Unclear	Clinic based	Simple random sampling	Observational Cross-sectional	EPDS>12	≥ Secondary vs. < secondary (ref) No association between education and postnatal depression (Bivariate, means comparison); p=0.13 Not working vs. working (ref) No association between employment and postnatal depression (Bivariate, means comparison); p=0.18
James-Hawkins, Shaltout, Nur, Nasrallah, Qutteina, Abdul Rahim, Hennink and Yount, 2019 [75]	Alharbi and Abdulghani, 2014 [80]	*352*	Saudi Arabia	PP	Depression	Unclear	Clinic based	Convenience sampling (non-probability sample)	Unclear	EPDS	≥ Secondary vs. < secondary (ref) No association between education and postnatal depression (Bivariate, chi square); p=0.79 Work or school vs. housewife(ref) No association between employment and postnatal depression (Bivariate, chi square), p=0.19
James-Hawkins, Shaltout, Nur, Nasrallah, Qutteina, Abdul Rahim, Hennink	Al Hinai and Al Hinai, 2014 [32]	*282*	Oman	PP: T1: 2 weeks T2: 8 weeks	Depression	Unclear	Hospital and health center	Unclear	Prospective cohort	EPDS	Work difficulties vs. none (ref) (among working women) No association between work difficulties and post-natal depression

and Yount, 2019 [75]											
James-Hawkins, Shaltout, Nur, Nasrallah, Qutteina, Abdul Rahim, Hennink and Yount, 2019 [75]	Bener, 2013 [81]	*1659*	Qatar	PP	Post-natal depression, anxiety and stress	Unclear	Clinic	Unclear	Systematic sampling (probability sample)	DASS-21	≥ Secondary vs. < secondary (ref) Negative association between education and postnatal depression (Bivariate, means comparison); p=0.01
James-Hawkins, Shaltout, Nur, Nasrallah, Qutteina, Abdul Rahim, Hennink and Yount, 2019 [75]	Bener, Burgut, Ghuloum and Sheikh, 2012 [74]	*1379*	Qatar	PP	Postnatal depression, anxiety and stress	Unclear	Clinic	Random sampling	Prospective cross sectional	EPDS	≥ Secondary vs. < secondary (ref) Negative association between education and postnatal depression (Bivariate, means comparison); p=0.00 Not working vs. Working(ref) No association between employment and postnatal depression (Bivariate, means comparison); p=0.12 Difficulty managing income vs. not (ref) Positive association between financial difficulties and postnatal depression (Multivariate, logistic regression); aOR = 2.37, p<0.001
James-Hawkins, Shaltout, Nur, Nasrallah, Qutteina,	Bener, Gerber and Sheikh, 2012 [23]	*1659*	Qatar	PP	Postnatal stress	Unclear	Clinic	Systematic sampling (probability sample)	Unclear	DASS-21	< Secondary vs. ≥ secondary (ref) Positive association between education and postnatal stress (Multivariate, logistic regression); aOR = 1.50, p=0.04

Abdul Rahim, Hennink and Yount, 2019 [75]												Positive association between education and postnatal depression (Multivariate, logistic regression); aOR = 1.50, p=0.01<br>Not working vs. working (ref)<br>Negative association between employment and postnatal depression (Multivariate, logistic regression); aOR = 1.6, p=0.00
James-Hawkins, Shaltout, Nur, Nasrallah, Qutteina, Abdul Rahim, Hennink and Yount, 2019 [75]	Burgut, et al., 2013 [82]	*1379*	Qatar	PP	Depression	Unclear	Clinic	Convenience sampling (non-probability sample)	Unclear	EPDS		< Secondary vs. ≥ secondary (ref)<br>No association between education and postnatal depression (Multivariate, logistic regression); Qatari: aOR = 1.62, p=0.08; Arab non-Qatari: aOR = 0.78, p=0.32<br>Working vs. not (ref)<br>No association between employment and postnatal depression (Multivariate, logistic regression); Qatari: aOR = 1.78, p= n.s.; Arab non-Qatari: aOR = 0.13, p= n.s
James-Hawkins, Shaltout, Nur, Nasrallah, Qutteina, Abdul Rahim, Hennink and Yount, 2019 [75]	Chaaya, Campbell, El Kak, Shaar, Harb and Kaddour, 2002 [25]	*396*	Lebanon	PP T1: 24 hrs T2: 3-5 months	Depression	Unclear	Hospitals, Community	Convenience	Prospective correlational	EPDS		Low and high vs. medium (ref)<br>No association between education and postnatal depression (Multivariate, logistic regression) low: OR = 1.12, p=0.77; high: OR =1.98, p=0.20<br>Working vs. not (ref)<br>No association between employment and postnatal depression (Multivariate, logistic regression); UaOR = 0.74, p=0.60

James-Hawkins, Shaltout, Nur, Nasrallah, Qutteina, Abdul Rahim, Hennink and Yount, 2019 [75]	El-Khoury, et al., 1999 [83]	*150*	Lebanon	PP	Depression	Unclear	Clinic	Convenience sampling (non-probability sample)	Unclear	DDI	≥ Secondary vs. < secondary (ref) No association between education and postnatal depression (Bivariate, means comparison); p=0.67
James-Hawkins, Shaltout, Nur, Nasrallah, Qutteina, Abdul Rahim, Hennink and Yount, 2019 [75]	Green, Broome and Mirabella, 2006 [26]	*T0= 125 T1= 86 T2= 56*	UAE	PP: T1: 3 months T2: 6 months	Depression	Unclear	Government maternity hospital	Convenience	Longitudinal, Prospective correlational	EPDS	Working vs not (ref) No association between employment and postnatal depression (Bivariate, means comparison); p>0.05
James-Hawkins, Shaltout, Nur, Nasrallah, Qutteina, Abdul Rahim, Hennink and Yount, 2019 [75]	Hamdan and Tamim, 2011 [27]	*137*	UAE	AP:2nd, 3rd trimesters PP T1: 2 months T2: 4 months	Depression	Unclear	Maternal and Child Health Centre	Convenience; two-stage sampling	Prospective longitudinal	Depressive symptoms-BDI Anxiety Symptoms-BAI Stressful life events Inventory MINI EPDS	> Secondary vs. ≤ secondary (ref) Negative association between education and postnatal depression (Bivariate, means comparison); p=0.04 Working vs not (ref) No association between employment and postnatal depression (Bivariate, means comparison); p=0.34
James-Hawkins, Shaltout,	Khabour, et al., 2013 [84]	*370*	Jordan	PP	Depression	Unclear	Clinic	Stratified Sampling	Unclear		≥ Secondary vs. < secondary (ref) No association between education and postnatal depression

Nur, Nasrallah, Qutteina, Abdul Rahim, Hennink and Yount, 2019 [75]								(probability sample)			(Bivariate, means comparison); p=0.32<br>≥ Secondary vs. < secondary (ref)<br>No association between education and postnatal depression (Bivariate, means comparison); p=0.32<br>Not satisfied with income vs. satisfied (ref)<br>Positive association between satisfaction with income and postnatal depression (Bivariate, means comparison); p=0.03
James-Hawkins, Shaltout, Nur, Nasrallah, Qutteina, Abdul Rahim, Hennink and Yount, 2019 [75]	Lteif, et al., 2005 [85]	*79*	Lebanon	AP: 1st, 2nd, 3rd trimesters	Depression	Unclear	Clinic	Convenience sampling (non-probability sample)	Unclear	BDI	Problems at work vs. Not (ref)<br>Positive association between problems at work and prenatal depression (Bivariate, logistic regression); UaOR=55.8, p=0.001
James-Hawkins, Shaltout, Nur, Nasrallah, Qutteina, Abdul Rahim, Hennink and Yount, 2019 [75]	Masmoudi, et al., 2014 [86]	*302*	Tunisia	PP	Depression	Unclear	Clinic	Convenience sampling (non-probability sample)	Unclear	EPDS	< Secondary vs. ≥ secondary (ref)<br>Positive association between education and postnatal depression (Bivariate, means comparison); p = 0.30 (PPD); p<0.01 (intense PPD)

James-Hawkins, Shaltout, Nur, Nasrallah, Qutteina, Abdul Rahim, Hennink and Yount, 2019 [75]	Masmoudi, et al., 2010 [87]	*213*	Tunisia	PP	Depression	Unclear	Clinic	Convenience sampling (non-probability sample)	Unclear	EPDS	Primary, secondary, post-secondary literate vs. illiterate (ref)<br>Negative association between education and postnatal depression (Bivariate, chi square); p = 0.03
James-Hawkins, Shaltout, Nur, Nasrallah, Qutteina, Abdul Rahim, Hennink and Yount, 2019 [75]	Masmoudi, Tabelsi, Charfeddine, Ben Ayed, Guermazzi and Jaoua, 2008 [35]	*213*	Tunisia	PP	Depression	Unclear	Clinic	Convenience sampling (non-probability sample)	Unclear	EPDS	≥ Secondary vs. < secondary (ref)<br>No association between education and postnatal depression (Bivariate, means comparison); p = 0.76
James-Hawkins, Shaltout, Nur, Nasrallah, Qutteina, Abdul Rahim, Hennink and Yount, 2019 [75]	Alami, Kadri and Berrada, 2006 [28]	*100*	Morocco	AP	Depression	Unclear	Clinic	Convenience sampling (non-probability sample)	Unclear	MINI	Illiterate vs. literate (ref)<br>No association between education and prenatal depression (Bivariate, logistic regression); p=0.93<br>Not working vs working (ref)<br>No association between employment and prenatal depression (Bivariate, logistic regression); p=0.68

James-Hawkins, Shaltout, Nur, Nasrallah, Qutteina, Abdul Rahim, Hennink and Yount, 2019 [75]	Alami, Kadri and Berrada, 2006 [28]	*100*	Morocco	PP	Depression	Unclear	Clinic	Convenien ce sampling (non-probability sample)	Unclear	MINI	Illiterate vs. literate (ref)<br>No association between education and perinatal depression (Bivariate, means comparison); p=0.90<br>Working vs. not (ref.)<br>No association between employment and perinatal depression (Bivariate, means comparison); p=0.20<br>Financial distress vs. none (ref.)<br>No association between financial distress and perinatal depression (Bivariate, means comparison); p=0.50
James-Hawkins, Shaltout, Nur, Nasrallah, Qutteina, Abdul Rahim, Hennink and Yount, 2019 [75]	Mohammad, Gamble and Creedy, 2011 [29]	*353*	Jordan	AP	Depression	Unclear	Clinic	Convenien ce sampling (non-probability sample)	Unclear	EPDS	Worry about financial problems<br>Positive association between financial problems and prenatal depression (Multivariate, regression); B=0.08, p=0.01
James-Hawkins, Shaltout, Nur, Nasrallah, Qutteina, Abdul Rahim, Hennink	Yehia, Callister and Hamdan-Mansour, 2013 [31]	*300*	Jordan	PP	Depression	Unclear	Clinic	Convenien ce sampling (non-probability sample)	Unclear	EPDS	Monthly income<br>Negative association between financial distress and postnatal depression (Multivariate, regression); B=-0.54, p=0.03

and Yount, 2019 [75]											
Jones and Coast, 2013 [37]	Husain, Bevc, Husain, Chaudhry, Atif and Rahman, 2006 [40]	*149/175 T1:175 (antenatal) T2: 149*	Pakistan	PP: 12week	Depression	Unclear	Rural community	Consecutively	Cross-sectional population-based survey	EPDS>=12 Social Support- MSPSS Mental Distress- SRQ-20	Domestic violence<br>Ua OR (95% CI) 2.9 (2.3-2.7)<br>Support assessed in postpartum period<br>Significant other p= 0.005<br>Low social support from family p= 0.021<br>Low social support from friends p= 0.014<br>Relationships assessed in postpartum period:<br>Marital problems<br>Ua OR (95% CI) 1.1 (0.5-2.2)<br>Relationship problems<br>Ua OR (95% CI) 0.6 (0.2-1.7)<br>Domestic violence<br>Ua OR (95% CI) 2.9 (2.3-2.7)
Jones and Coast, 2013 [37]	Rahman, Iqbal and Harrington, 2003 [39]	*541/632*	Pakistan	PP: 10-12 weeks	Depression	Unclear	Rural Community	Convenience	Longitudinal	Clinical assessment in neuropsychiatry (ICD-10 diagnosis)	Household and family structure:<br>Living in extended family<br>Ua OR (95% CI) 0.6 (0.4-0.8)<br>Living in nuclear family<br>Aa OR (95% CI) 4.3 (1.4-13.3) p< 0.05<br>Infant's grandmother lives with family<br>Ua OR (95% CI) 0.6 (0.5-0.8)<br>Husband away for >6 months<br>Ua OR (95% CI) 0.9 (0.7-1.4)<br>Support assessed in postpartum period:<br>Daily Support in childcare by at least one family member<br>Ua OR (95% CI) 0.5 (0.4-0.7)<br>Aa OR (95% CI) 0.3 (0.1-0.7)

											Able to complete *chilla* period<br>Ua OR (95% CI) 0.4 (0.3-0.6)<br>Aa OR (95% CI) 0.3 (0.2-0.7)<br>Relationship assessed during pregnancy:<br>Serious arguments with family<br>Aa 4.4 (1.9-10.3) Ua Not reported
Jones and Coast, 2013 [37] Klainin and Arthur, 2009 [24]	Rahman and Creed, 2007 [41]	*160*	Pakistan	Followed up women diagnosed with prenatal depression to one year postpartum. Dependent variable was persistence of depression at 3, 6, 12 months postpartum. 56.6% followed up to 12 months had	Depression	Unclear	Rural Community	Convenience	Longitudinal	Clinical assessment in neuropsychiatry (ICD-10 diagnosis)	Support assessed during pregnancy:<br>Lack of social support<br>Ua OR (95% CI) 0.9 (0.7-1.3)<br>Household and family structure:<br>Husband away for>6 months<br>Ua OR (95% CI) 0.9 (0.7-1.4)<br>Living in nuclear family<br>Ua OR (95% CI) 1.2 (0.9-2.0)<br>Relationships assessed during pregnancy<br>Serious marital problems<br>Ua OR (95% CI) 1.0 (0.5-1.9)<br>Serious arguments with family<br>Ua OR (95% CI) 1.3 (0.9-1.8)<br>Loss of friend or confidant<br>Ua OR (95% CI) 1.5 (1.1-1.9)<br>Not significant Aa association for multivariate analysis

				persistent depression.							
Klainin and Arthur, 2009 [24]	Chaaya, Campbell, El Kak, Shaar, Harb and Kaddour, 2002 [25]	*396*	Lebanon	PP: 7 days, 4-5 months	Depression	Unclear	Hospitals, Community	Convenience	Prospective correlational	EPDS	Poor social support<br>Antenatal depression<br>Chronic health problems<br>Stressful life events<br>Vaginal delivery<br>Low education<br>Unemployment
Klainin and Arthur, 2009 [24]	Green, Broome and Mirabella, 2006 [26]	*T0= 125*<br>*T1= 86*<br>*T2= 56*	UAE	PP: few days, 3, 6 months	Depression	Unclear	Government maternity hospital	Convenience	Longitudinal, Prospective correlational	EPDS>13	The absence of breast feeding<br>Women who are unable to follow religious expectation (breastfeed for at least 2 years) may experience internal guilt, which may contribute to tremendous stress and thus increase a possibility of depressive symptoms<br>Premiparae<br>Poor self-image<br>Marriage at older age<br>Poor relationships with mother-in-law
Klainin and Arthur, 2009 [24]	Husain, Bevc, Husain, Chaudhry, Atif and Rahman, 2006 [40]	*T1: 175 (antenatal)*<br>*T2: 149*	Pakistan	PP: 3 months	Depression	Unclear	Rural community	Convenience	Prospective correlational	EPDS>=12	Domestic violence<br>Low social support from significant others, friends, and family<br>Antenatal psychological problems
Klainin and Arthur, 2009 [24]	Stuchbery, et al., 1998 [88]	*Unclear*	Unclear	Unclear	Unclear	Unclear	Unclear	Unclear	Unclear	Unclear	In Arab cultures, family connotes a centre of honour, loyalty, and reputation with a large extended family is greatly appreciated.<br>Women are traditionally

											subordinate to male relatives; however, they receive the recognition of honour and substantial social support during a 40-day postpartum period
Klainin and Arthur, 2009 [24]	Kalyani, Saeed, Rehman and Mubbashar, 2001 [42]	*T1: 120 T2: 76*	Pakistan	PP: T1: 2 weeks T2: 2-4 weeks	Depression	Unclear	Home visit, Community	Convenience	Prospective comparative	EPDS >10	Unwanted pregnancy<br>Parity (Premiparae)<br>Living with extended family<br>Marital difficulties<br>Early loss of mother
Qutteina, et al., 2018 [89]	Abdelhai and Mosleh, 2015 [76]	*376*	Egypt	AP: 1st, 2nd, 3rd trimester	Prenatal depression and anxiety	Unclear	Clinic	Systematic sampling (probability sample)	observational Cross-sectional	Unclear	Spouse domestic violence is an independent predictor of prenatal depression and anxiety combined (OR = 3.27 p = 0.013; Binary logistic regression)<br>Problematic familial relationships are not significant predictors of prenatal depression and anxiety combined (OR = 2.02 p = 0.08; Binary logistic regression)
Qutteina, Nasrallah, James-Hawkins, Nur, Yount, Hennink and Abdul Rahim, 2018 [89]	Abujilban, Abuidhail, Al-Modallal, Hamaideh and Mosemli, 2014 [78]	*218*	Jordan	AP	Depression	Unclear	Clinic	Convenience sampling (non-probability sample)	Observational Cross sectional	DUCOS	Family and Nonfamily stressful relationships are significant predictors of prenatal depression (B = 0.08 p < 0.01 B = 0.08 p < 0.01; Multiple regression).<br>Social support from family and non-family resources was not associated with decreased prenatal depression (B = not reported p > 0.05; Multiple regression)
Qutteina, Nasrallah, James-Hawkins, Nur, Yount,	Abou-Saleh and Ghubash, 1997 [17]	*90*	UAE	PP	Depression	Unclear	Unclear	Census of setting	Observational Prospective	Unclear	Marital problems before birth (Yes: 5 case, 2 non case No: 10 case, 69 non case; p = 0.001) and ongoing (Yes: 5 case, 9 non case No: 10 case, 63 non case p = 0.05; Bivariate, contingency tables)

Hennink and Abdul Rahim, 2018 [89]											are significantly positively associated with PPD. Decreased family support (Staying with women's family (not in-laws)) is a significant predictor of PPD ($p < 0.001$; DFA (Discriminant Function Analysis)) Husband assistance is significantly inversely associated with PPD (Yes: 9 case, 55 non case No: 7 case, 18 non case $p = 0.05$)
Qutteina, Nasrallah, James-Hawkins, Nur, Yount, Hennink and Abdul Rahim, 2018 [89]	Agoub, Moussaoui and Battas, 2005 [22]	*144*	Morocco	PP: 2 and 6 weeks, 6 and 9 months	Depression	Unclear	Primary healthcare centres	Consecutiv e	Longitud inal	MINI	Poor marital relationship is significantly associated with PPD (33.3% case, 13.6% non - case $p = 0.02$; Bivariate, ANOVA)
Qutteina, Nasrallah, James-Hawkins, Nur, Yount, Hennink and Abdul Rahim, 2018 [89]	Alasoom and Koura, 2014 [90]	*450*	Saudi Arabia	PP	Depression	Unclear	Clinic/ Hospital	Census	Observat ional cross-sectional	Unclear	Non-supportive husband is a significant independent predictor of PPD (OR 2.493; $p = 0.011$; Stepwise logistic regression) Poor support from relatives (Non-supportive relatives) is not a significant predictor of PPD (OR = not reported $p > 0.005$; Stepwise logistic regression)
Qutteina, Nasrallah, James-Hawkins, Nur,	Al-Azri, Al-Lawati, Al-Kamyani, Al-Kiyumi, Al-Rawahi,	*959*	Oman	AP	Depression	Unclear	Clinic	Unclear	Observat ional cross-sectional	Unclear	Marital conflict is a significant predictor of prenatal depression (OR = 13.83 $p = 0.000$; Multivariate logistic regression)

Yount, Hennink and Abdul Rahim, 2018 [89]	Davidson and Al-Maniri, 2016 [79]										
Qutteina, Nasrallah, James-Hawkins, Nur, Yount, Hennink and Abdul Rahim, 2018 [89]	Al Dallal and Grant, 2012 [73]	*237*	Bahrain	PP: 8 weeks	Depression	Unclear	Clinic based	Simple random sampling	Observational Cross-sectional	EPDS>12	Non-supportive husband is a significant predictor of PPD (OR 2.41 p = 0.01; Multivariate logistic regression)<br>Less help provided by mother is not significantly associated with PPD (OR = 1.33 p = 0.292; Bivariate, Chi-square)<br>Less satisfaction with help at home is significantly associated with increased PPD (OR = 2.00 p = 0.04; Bivariate)
Qutteina, Nasrallah, James-Hawkins, Nur, Yount, Hennink and Abdul Rahim, 2018 [89]	Al Hinai and Al Hinai, 2014 [32]	*282*	Oman	PP: T1: 2 weeks<br>T2: 8 weeks	Depression	Unclear	Hospital and health center	Unclear	Prospective cohort	EPDS>=13<br><br>EPDS 10-12	Conflict with a family member is a significant predictor of PPD (at 2 weeks, but not at 8 weeks) (At 2 weeks: OR = 1.7, p = 0.017; At 8 weeks: OR = 1.468, p = 0.229; Binomial regression)
Qutteina, Nasrallah, James-Hawkins, Nur, Yount, Hennink and Abdul	Alami, Kadri and Berrada, 2006 [28]	*100*	Morocco	PP: 2-3 weeks<br>12 weeks<br>24 weeks<br>36 weeks	Depression	Unclear	Clinic/ Hospital	Unclear	Observational, Prospective cohort study	MINI (DSM-IV)	Poor relationship with spouse significantly associated with PPD (14.2% case, 22.6% noncase p < 0.001; Bivariate, ANOVA)

Rahim, 2018 [89]											
Qutteina, Nasrallah, James-Hawkins, Nur, Yount, Hennink and Abdul Rahim, 2018 [89]	Bener, Burgut, Ghuloum and Sheikh, 2012 [74]	*1379*	Qatar	PP	Postnatal depression, anxiety and stress	Unclear	Clinic/ Hospital	Systematic Random sampling	Observational cross sectional	Unclear	Dissatisfaction in married life is a significant predictor of PPD (OR = 1.26 p = 0.005) Poor marital relationship is a significant predictor of PPD (OR = 1.13 p = 0.048; Multivariate logistic regression) A bad relationship with mother-in-law is significantly associated with PPD (Good: 29.6% case, 43% non-case Bad: 70.4% case, 57% non-case P < 0.001; Bivariate, Chi-square). Poor family support is a significant predictor of PPD (OR = 1.52 p = 0.016; Multivariate logistic regression)
Qutteina, Nasrallah, James-Hawkins, Nur, Yount, Hennink and Abdul Rahim, 2018 [89]	Bener, Gerber and Sheikh, 2012 [23]	*1659*	Qatar	PP	Anxiety, Stress, PPD (Postpartum Depression)	Unclear	Unclear	Systematic random sampling	Observational Cross-sectional	Unclear	Dissatisfaction in married life is a significant predictor of postpartum anxiety and stress. It is not a significant predictor of PPD (OR = 1.6 p = 0.02; Multivariate logistic regression) Relationship with mother-in-law is not a significant predictor of PPD (OR = not reported p > 0.05), anxiety (OR = not reported p > 0.05) or stress (OR = not reported p > 0.05; Multivariate logistic regression) Poor family support (Lack of family support) is a significant predictor of PPD (OR = 1.6 p = 0.005) and anxiety (OR = 1.9 p < 0.001). It is not a significant

											predictor of stress (OR = not reported p> 0.005; Multivariate logistic regression)
Qutteina, Nasrallah, James-Hawkins, Nur, Yount, Hennink and Abdul Rahim, 2018 [89]	Chaaya, Campbell, El Kak, Shaar, Harb and Kaddour, 2002 [25]	*396*	Lebanon	PP	Depression	Unclear	Unclear	Census of setting	Observational Prospective cohort	Unclear	Poor social support (Social support (more than confidant)) is not a significant predictor of PPD (OR = 0.66 p > 0.05; Multiple logistic regression)
Qutteina, Nasrallah, James-Hawkins, Nur, Yount, Hennink and Abdul Rahim, 2018 [89]	El-Hachem, et al., 2014 [91]	*149*	Lebanon	PP	Depression	Unclear	Unclear	Convenience sampling	Observational Cross-sectional	MSPSS for poor social support	Lack of family support is not a significant predictor of PPD (OR = not reported p > 0.05; Stepwise multiple regression)
Qutteina, Nasrallah, James-Hawkins, Nur, Yount, Hennink and Abdul Rahim, 2018 [89]	Ghubash and Abou-Saleh, 1997 [92]	*95*	UAE	PP	Depression	Unclear	Clinic/ Hospital	Census of setting	Observational Prospective cohort	Unclear	Marital problems before birth are a significant predictor of PPD (p < 0.0001; DFA (Discriminant Function Analysis))
Qutteina, Nasrallah, James-	Green, Broome and	*3 months: 86*	UAE	PP: 3,6 months	Depression	Unclear	Clinic/ Hospital	Census of setting	Observational	Unclear	Relationship with husband is not significantly associated with PPD (p= n. s.; Bivariate, Chi-square).

Hawkins, Nur, Yount, Hennink and Abdul Rahim, 2018 [89]	Mirabella, 2006 [26]	*6 months: 56*							Prospective cohort		Relationship with own mother is not significantly associated with PPD (p= n. s. Bivariate, Chi-square). Relationship with mother-in-law is significantly associated with PPD (at 3 months, but not at 6 months) (At 3 months: p = 0.02, at 6 months: p= n. s.; Bivariate)
Qutteina, Nasrallah, James-Hawkins, Nur, Yount, Hennink and Abdul Rahim, 2018 [89]	Lteif, Kesrouani and Richa, 2005 [85]	*79*	Lebanon	AP	Depression	Unclear	Clinic/ Hospital	Census of setting	Observational Cross-sectional	Unclear	Marital problems (OR not reported p = 0.018; Bivariate, logistic regression), poor husband support (OR = 10.4 p = 0.001; Bivariate, logistic regression) , poor quality of sexual relationship prior to pregnancy (OR = 13.3 p = 0.001; Bivariate, logistic regression) and decreased desire of the husband (OR not reported p > 0.05; Bivariate, logistic regression) are significantly associated with prenatal depression. Poor quality of sexual relationship during pregnancy (OR = 6.9 p = 0.002; Bivariate, logistic regression) is not significantly associated with prenatal depression. Poor family support is not significantly associated with prenatal depression (OR not reported p > 0.05; Bivariate, logistic regression)
Qutteina, Nasrallah, James-Hawkins, Nur,	Masmoudi, Tabelsi, Charfeddine , Ben Ayed, Guermazzi	*2–5 days: 213*	Tunisia	PP:2-5 days, 6-8 weeks	Depression	Unclear	Clinic/ Hospital	Census of setting	Observational Prospective cohort	Unclear	Poor quality of marital relationship is significantly associated with PPD (Good: 73.52% case, 11.02% non-case Bad: 13.23% case, 2.2% non-

Yount, Hennink and Abdul Rahim, 2018 [89]	and Jaoua, 2008 [35]	*6–8 weeks: 136*									case p = 0.034; Bivariate, Chi-square)
Qutteina, Nasrallah, James-Hawkins, Nur, Yount, Hennink and Abdul Rahim, 2018 [89]	Masmoudi, Charfeddine, Trabelsi, Feki, Ben Ayad, Guermazi, Baati and Jaoua, 2014 [86]	*2–5 days: 302 6–10 weeks: 139*	Tunisia	PP: 2-5 days,6-10 weeks	Depression	Unclear	Clinic/ Hospital	Census of setting	Observational Prospective cohort	MSSS for poor social support	Lower quality of marital relationship (AZRIN is significantly associated with PPD (p = 0.034; Bivariate, Chi-square) Poor social support is not significantly associated with PPD (p = 0.29; Bivariate, Chi-square)
Qutteina, Nasrallah, James-Hawkins, Nur, Yount, Hennink and Abdul Rahim, 2018 [89]	Masmoudi, Trabelsi, Charfeddine, Ben Ayed, Guermazi, Jaoua, Karam and Hantouche, 2010 [87]	*213*	Tunisia	PP	Depression	Unclear	Clinic/ Hospital	Census of setting	Observational cross-sectional	Unclear	Poor quality of marital relationship is a significant predictor of PPD (OR = 3.806 p = 0.009; Multivariate logistic regression). Poor family support (Lack of social and family support) is a significant predictor of PPD (OR = 2.265 p = 0.071; Multivariate logistic regression)
Qutteina, Nasrallah, James-Hawkins, Nur, Yount, Hennink and Abdul Rahim, 2018 [89]	Mohammad, Gamble and Creedy, 2011 [29]	*353*	Jordan	PP	Depression	Unclear	Hospital	Unclear	Observational cross-sectional	MSSS for poor social support	Difficult marital relationship is significantly associated with PPD (At 6–8 weeks: p = 0.009; At 6 months: p = 0.003; Bivariate, Chi-square). Difficult relationship with mother-in-law is a significant predictor of PPD (B =-0.194 p < 0.001; Stepwise multiple regression). Poor social support score is a significant predictor of PPD (B = -

											0.123 p = 0.003; Stepwise multiple regression)
Qutteina, Nasrallah, James-Hawkins, Nur, Yount, Hennink and Abdul Rahim, 2018 [89]	Nayak and Al-Yattama, 1999 [93]	*248*	Kuwait	AP	Depression	Unclear	Clinic/ Hospital	Census of setting	Cross-sectional	Unclear	Marital conflict is not significantly associated with prenatal depression ($p = 0.647$; Analysis of covariance) Past assault history (familial or non-familial) is significantly associated with prenatal depression ($p < 0.001$; Analysis of covariance)
Qutteina, Nasrallah, James-Hawkins, Nur, Yount, Hennink and Abdul Rahim, 2018 [89]	Osman, et al., 2014 [94]	*452*	Lebanon	PP	Postpartum Stress	Unclear	Unclear	Census of setting	Randomized controlled trial (Single blinded)	Unclear	Increased social support is significantly associated with lower postpartum stress: Postpartum support film alone (Treatment ((15.76 ± 6.55) vs control group (18.93 ± 7.03) $p < 0.01$)) Postpartum support film with hotline service (Treatment (15.86 ± 6.81) vs control group (18.93 ± 7.03) $p < 0.01$)) Hotline service alone (Treatment (16.98± 6.42) vs control group (18.93 ±7.03) $p < 0.05$) Bivariate test
Qutteina, Nasrallah, James-Hawkins, Nur, Yount, Hennink and Abdul Rahim, 2018 [89]	Yehia, Callister and Hamdan-Mansour, 2013 [31]	*300*	Jordan	PP	Depression	Unclear	Clinic/ Hospital	Convenience sampling	Observational Cross-sectional	MSPSS	Poor family social support is a significant predictor of PPD (B = -0.13 p = 0.007; Multiple hierarchical regression). Poor friend (B = not reported $p > 0.05$; Multiple hierarchical regression) and others social support is not a significant predictor of PPD (B = not reported $p > 0.05$; Multiple hierarchical regression).

Sawyer, Ayers and Smith, 2010 [20]	Alami, Kadri and Berrada, 2006 [28]	*100*	Morocco	Age (19-43); AP: 1st,2nd and 3rd trimester	Depression	Unclear	Unclear	Unclear	Longitud inal		Non-significant factors:<br>Age<br>Education<br>Parity
Sawyer, Ayers and Smith, 2010 [20]	Alami, Kadri and Berrada, 2006 [28]	*100*	Morocco	PP: 2-3 weeks<br>12 weeks,<br>24 weeks,<br>36 weeks	Depression	Unclear	Unclear	Unclear	Longitud inal	MINI (DSM-IV)	Significant risk Factors:<br>Complications during pregnancy<br>Low birth weight<br>Marital conflict<br>Unhappiness during pregnancy<br>Non-significant risk factors:<br>Age<br>Education<br>Employment<br>Parity<br>Mode of delivery<br>Place of delivery
Sawyer, Ayers and Smith, 2010 [20]	Agoub, Moussaoui and Battas, 2005 [22]	*144*	Morocco	PP: 2-3 weeks<br>6 weeks<br>6th month,<br>9th month<br>Age (18-44);	Depression	Unclear	Unclear	Unclear	Longitud inal	MINI (DSM-IV)	Significant risk Factors:<br>Complications during pregnancy<br>Marital conflict<br>Lack of support from family and partner<br>Non-significant risk factors:<br>Age<br>Education<br>Employment<br>Marital status<br>Mode of delivery<br>Place of delivery
Shorey, Chee, Ng, Chan, Tam and Chong, 2018 [21]	Agoub, Moussaoui and Battas, 2005 [22]	*144*	Morocco	PP: 2-3 weeks<br>6 weeks<br>6th month,<br>9th month	Depression	Unclear	primary healthcare setting	Convenien ce	Prospecti ve cohort	MINI<br>EPDS (Arabic version)	Pregnancy complications<br>Stressful life events during pregnancy<br>Baby's health problems<br>Poor marital relationship

Shorey, Chee, Ng, Chan, Tam and Chong, 2018 [21]	Al Hinai and Al Hinai, 2014 [32]	*282*	Oman	PP: 2 weeks, 8 weeks	Depression	Unclear	Primary health care facility	Unclear	Prospective cohort	EPDS>=13	Work difficulties<br>Conflict with family member<br>Sickness of a family member
Shorey, Chee, Ng, Chan, Tam and Chong, 2018 [21]	Green, Broome and Mirabella, 2006 [26]	*56*	UAE	PP: 3, 6 months	Depression	Unclear	Government maternity hospital	Convenience	Prospective cohort	EPDS>13 (Arabic version)	Not breastfeeding<br>First childbirth<br>Poor self-body image and view of weight<br>Poor relationship with mother-in-law<br>Older age at marriage
Shorey, Chee, Ng, Chan, Tam and Chong, 2018 [21]	Hamdan and Tamim, 2011 [27]	*137*	UAE	AP: 2nd, 3rd trimester<br>PP:2 months	Depression	Unclear	maternal and child health center	Convenience	Prospective cohort	Depressive symptoms-BDI<br>Anxiety Symptoms-BAI<br>Stressful Life events-Stressful life events Inventory<br>Postpartum Depression<br>-MINI (Diagnosis)<br>-EPDS (>10) (Screening)	Depression during pregnancy<br>Number of children<br>Religion<br>Low educational level of mother<br>Lack of breastfeeding<br>Stressful life events<br>Employment status following delivery
Shorey, Chee, Ng, Chan, Tam and Chong, 2018 [21]	Husain, Bevc, Husain, Chaudhry, Atif and Rahman, 2006 [40]	*149*	Pakistan	PP: 12 weeks	Depression	Unclear	Community	Convenience	Cross-sectional population-based survey	EPDS<br>Social Support-MSPSS<br>Mental Distress- SRQ-20	Lower social support<br>Increased stressful life events in the preceding year<br>Higher levels of psychological distress in the antenatal period.

Shorey, Chee, Ng, Chan, Tam and Chong, 2018 [21]	Rahman, Iqbal and Harrington, 2003 [39]	*541*	Pakistan	AP: 3rd trimester PP: 10-12 weeks	Depression	Unclear	Community	Unclear	Unclear	SCAN PIQ LEDS BDQ	More disabilities<br>More threatening life events<br>Poorer social and family support<br>Vulnerable mothers were more likely to be depressed during pregnancy, rather than have an onset in the post-natal period
Shorey, Chee, Ng, Chan, Tam and Chong, 2018 [21]	Rahman and Creed, 2007 [41]	*129*	Pakistan	AP: 3rd trimester PP: 3, 6, 12 months	Depression	Unclear	Unclear	Unclear	Prospective study	SCAN PIQ LEDS BDQ	Poverty<br>Having 5 or more children<br>Uneducated husband<br>Lack of a confidant or friend
Zahidie and Jamali, 2013 [38]	Ali, Ali and Azam, 2009 [58]	*Unclear*	Pakistan	PP: 1,2,6 and 12 months	Depression	Unclear	Urban communities of karachi	Unclear	Quasi-experimental study	AKUADS DSM IV	Domestic violence<br>Difficulty in breast feeding at birth (p-value = 0.125)<br>Child having any difficulty soon after birth (cyanosis/apnea/flaccidity, p-value = 0.03)<br>Unplanned pregnancy (p-value = 0.038)
Zahidie and Jamali, 2013 [38]	Husain, Bevc, Husain, Chaudhry, Atif and Rahman, 2006 [40]	*Unclear*	Pakistan	AP, PP- 3 months	Depression	Unclear	community setting of Kallar Syedan, one of four administrative Circles of a sub-district in Rawalpindi	Unclear	Population-based survey	EPDS MSPSS PIQ	Lower social support<br>Increased stressful life events in the preceding year<br>Higher levels of psychological distress in the antenatal period
Zahidie and Jamali, 2013 [38]	Husain, Parveen, Husain, Saeed, Jafri,	*Unclear*	Pakistan	AP	Depression	Unclear	antenatal clinics of Chiniot and	Unclear	Cohort study	EPDS	Disability and stressful life events.

	Rahman, Tomenson and Chaudhry, 2011 [47]						maternity and childcare centers				
Zahidie and Jamali, 2013 [38]	Husain, Parveen, Husain, Saeed, Jafri, Rahman, Tomenson and Chaudhry, 2011 [47]	*149*	Pakistan	PP: 12 weeks	Depression	Unclear	antenatal clinics of Chiniot and maternity and childcare centers	Unclear	Cohort study	EPDS	Disability and stressful life events.
Zahidie and Jamali, 2013 [38]	Asad, Karmaliani, Sullaiman, Bann, McClure, Pasha, Wright and Goldenberg, 2010 [59]	*1368*	Pakistan	AP	Depression / Anxiety	Unclear	Primary care health centres	Unclear	Cross sectional study	AKUADS	Verbal or physical/sexual abuse
Zahidie and Jamali, 2013 [38]	Asad, Karmaliani, Sullaiman, Bann, McClure, Pasha, Wright and Goldenberg, 2010 [59]	*148*	Pakistan	AP	Attempted Suicide	Unclear	Primary care health centres	Unclear	Cross sectional study	AKUADS	Verbal or physical/sexual abuse
Zahidie and Jamali, 2013 [38]	Rahman, Iqbal and Harrington, 2003 [39]	*632*	Pakistan	AP: 3rd trimester	Depression	Unclear	Unclear	Unclear	Two stage cross-	SCAN PIQ BDQ ICD-10	Disability Threatening life events Poor social and family support.

									sectional survey		
Zahidie and Jamali, 2013 [38]	Rahman, Iqbal and Harrington, 2003 [39]	*541*	Pakistan	PP:10-12 weeks post delivery	Depression	Unclear	Unclear	Unclear	Two stage cross-sectional survey	SCAN<br>PIQ<br>BDQ<br>ICD-10	Disability<br>Threatening life events<br>Poor social and family support<br>Financial independence of women.<br>No association between post-natal depression and husband's monthly income, or poor socioeconomic situation
Zahidie and Jamali, 2013 [38]	Rahman and Creed, 2007 [41]	*Unclear*	Pakistan	AP:3, 6 months	Depression	Unclear	Unclear	Unclear	Four stage cross-sectional survey	Unclear	poverty during pregnancy
Zahidie and Jamali, 2013 [38]	Rahman and Creed, 2007 [41]	*Unclear*	Pakistan	PP: 3, 12 months	Depression	Unclear	Unclear	Unclear	Four stage cross-sectional survey	Unclear	poverty during pregnancy
Zahidie and Jamali, 2013 [38]	Zahidie, Kazi, Fatmi, Bhatti and Dureshahwar, 2011 [53]	*Unclear*	Pakistan	AP	Depression	Unclear	Rural community	Unclear	Mix method study. Qualitative phase I: (in-depth interviews) Quantitative phase II: (cross-sectional survey)	CEDS	Social conditions, as compared to social relations, were more important determinants for depression among women. The results of this study showed that for each unit increase in poor social conditions, there was 0.57 increase in depression scores.
Additional primary	Husain, Munshi,	*1357*	Pakistan	AP: 3rd trimester	Depression	March 2004 to	Maternity home and	Consecutive	Cohort study	PIQ	No significant association with Low birth weight in newborns

studies identified	Jafri, Husain, Parveen, Saeed, Tomenson, Naeem and Chaudhry, 2014 [61]					April 2005	childcare center			EPDS- Urdu version SRQ-20	Problem with previous delivery 0.877 (0.728-1.056)
	Amr and Hussein Balaha, 2010 [95]	*367*	Saudi Arabia	Teenage and Primigravid mothers	Perinatal depression	Unclear	Unclear	Unclear	Unclear	MINI	Urban residence<br>Poor husband support<br>History of psychiatric illness<br>Anaemia<br>Caesarean delivery<br>Female delivery
	Ghubash and Eapen, 2009 [96]	*19*	UAE	AP	Perinatal depression	Unclear	Unclear	Public and private clinic	Unclear	Focus groups	Difficult delivery<br>Unhealthy baby<br>Difficult baby and poor bonding<br>Lack of support<br>Loneliness and isolation from family of origin<br>Conflict with regards to mother's role
	Al-Modayfer, Alatiq, Khair and Abdelkawi, 2015 [63]	*571*	Saudi Arabia	PP: 5 weeks after delivery	Depression	Unclear	Community	Unclear	Unclear	EPDS	Previous psychiatric history<br>Mother's health during pregnancy<br>Delivery time

(SSDS) The Siddique Shah Depression scale; (EPDS) Edinburgh Postnatal Depression Scale; (PDSS)Postnatal Depression Screening scale; (BDI)Beck Depression Inventory; (HADS) Hospital Anxiety and Depression Scale; (DASS) Depression Anxiety Stress Scale; (BDQ) Brief Disability Questionnaire; (PIQ)Personal Information Questionnaire; (LEDS) Life events and difficulties Schedule; (BAI) Anxiety Symptoms; (DUCOS)Duke Social Support and Stress Scale; (MSSS) Maternity Social Support Score; (DDI) Depression Detailed Inventory; (PKS) Perceived knowledge scale; (PSES) Perceived Self-efficacy scale; (SRQ-20) Self Reporting Questionnaire; (SCAN) WHO Schedule for Clinical Assessment in Neuropsychiatry; (MSPSS) Multidimensional Scale of Perceived Social Support; (CES-D) The Centre for Epidemiology Studies-Depression scale; (AKUADS) Aga khan University Anxiety Depression Scale AP- Antepartum PP- Postpartum T- Time frame PND- Perinatal Depression PPD- Postpartum Depression ref-Reference UaA- Unadjusted association Aa- adjusted association CI-Confidence Interval

Table S7: Quality assessment of prevalence outcomes in Middle East and North Africa

Systematic Review	Primary study	Population	Outcome	Data collection time	Study setting
Afzal and Khalid, 2016 [4]	Rasheed, 1988 [5]	Yes	Yes	No	No
Afzal and Khalid, 2016 [4]	Khalid, 1989 [6]	Yes	Yes	No	No
Afzal and Khalid, 2016 [4]	Sarwar, 1990 [7]	Yes	Yes	No	No
Afzal and Khalid, 2016 [4]	Habib, 1997 [8]	Yes	Yes	No	No
Afzal and Khalid, 2016 [4]	Ahmed, 2005 [9]	Yes	Yes	No	Yes
Afzal and Khalid, 2016 [4]	Munaf, 2006 [71]	Yes	Yes	No	No
Afzal and Khalid, 2016 [4]	Muneer, Minhas, Tamiz-ud-Din Nizami, Mujeeb and Usmani, 2009 [10]	Yes	Yes	No	No
Afzal and Khalid, 2016 [4]	Karmaliani, Asad, Bann, Moss, McClure, Pasha, Wright and Goldenberg, 2009 [12]	Yes	Yes	No	No
Afzal and Khalid, 2016 [4]	Haider, 2010 [13]	Yes	Yes	No	No
Afzal and Khalid, 2016 [4]	Yasmeen, Tayyaba, Chatan, Naeem, Numan and Adnan Maqsood, 2010 [14]	Yes	Yes	No	Yes
Afzal and Khalid, 2016 [4]	Shah, Bowen, Afridi, Nowshad and Muhajarine, 2011 [15]	Yes	Yes	No	No
Afzal and Khalid, 2016 [4]	Gul, Bajwa, Niaz, Haroon, Liaqat, Ahmad, Dawood, Ghazal, Bhatti, Nazir, Riffat and Chaudhry, 2013 [70]	Yes	Yes	No	No
Afzal and Khalid, 2016 [4]	Afzal and Khalid, 2014 [69]	Yes	Yes	No	No
Afzal and Khalid, 2016 [4]	Ghazala Sadiq, 2015 [72]	Yes	Yes	No	No
Alhasanat and Fry-McComish, 2015 [16]	Saleh el, El-Bahei, Del El-Hadidy and Zayed, 2013 [30]	Yes	Yes	No	No
Amber Haque, 2015 [18]	Balaha, Amr, El-Gilany and Sheikh, 2009 [34]	Yes	Yes	Unclear	Yes
Amber Haque, 2015 [18]	Naglaa A. Mohamed and Maklof, 2011 [33]	Yes	Yes	No	Yes
Fisher, Cabral de Mello, Patel, Rahman, Tran, Holton and Holmes, 2012 [19]	Karmaliani, Bann, Mahmood, Harris, Akhtar, Goldenberg and Moss, 2006 [36]	Yes	Yes	No	Yes
Fuhr, Calvert, Ronsmans, Chandra, Sikander, De Silva and Patel, 2014 [64]	Farhat, Chaouch, Chelli, Gara, Boukraa, Garbouj, Hamrouni, Fourati, Calvez and Thonneau, 2012 [65]	Yes	Yes	Yes	Yes
Fuhr, Calvert, Ronsmans, Chandra, Sikander, De Silva and Patel, 2014 [64]	Jafarey, Rizvi, Koblinsky and Kureshy, 2009 [67]	Yes	Yes	Yes	Yes

Fuhr, Calvert, Ronsmans, Chandra, Sikander, De Silva and Patel, 2014 [64]	Population, 2001 [68]	Yes	Yes	Yes	Yes
Fuhr, Calvert, Ronsmans, Chandra, Sikander, De Silva and Patel, 2014 [64]	Amarin, Khader, Okour, Jaddou and Al-Qutob, 2010 [66]	Yes	Yes	Yes	Yes
James-Hawkins, Shaltout, Nur, Nasrallah, Qutteina, Abdul Rahim, Hennink and Yount, 2019 [75]	Abuidhail and Abujilban, 2014 [77]	Yes	Yes	Unclear	Yes
James-Hawkins, Shaltout, Nur, Nasrallah, Qutteina, Abdul Rahim, Hennink and Yount, 2019 [75]	Al Dallal and Grant, 2012 [73]	Yes	Yes	Unclear	Yes
James-Hawkins, Shaltout, Nur, Nasrallah, Qutteina, Abdul Rahim, Hennink and Yount, 2019 [75]	Alharbi and Abdulghani, 2014 [80]	Yes	Yes	Unclear	Yes
James-Hawkins, Shaltout, Nur, Nasrallah, Qutteina, Abdul Rahim, Hennink and Yount, 2019 [75]	Al Hinai and Al Hinai, 2014 [32]	Yes	Yes	Unclear	Yes
James-Hawkins, Shaltout, Nur, Nasrallah, Qutteina, Abdul Rahim, Hennink and Yount, 2019 [75]	Bener, 2013 [81]	Yes	Yes	Unclear	Yes
James-Hawkins, Shaltout, Nur, Nasrallah, Qutteina, Abdul Rahim, Hennink and Yount, 2019 [75]	Burgut, Bener, Ghuloum and Sheikh, 2013 [82]	Yes	Yes	Unclear	Yes
James-Hawkins, Shaltout, Nur, Nasrallah, Qutteina, Abdul Rahim, Hennink and Yount, 2019 [75]	El-Khoury, Karam and Melhem, 1999 [83]	Yes	Yes	Unclear	Yes
James-Hawkins, Shaltout, Nur, Nasrallah, Qutteina, Abdul Rahim, Hennink and Yount, 2019 [75]	Hamdan and Tamim, 2011 [27]	Yes	Yes	Unclear	Yes
James-Hawkins, Shaltout, Nur, Nasrallah, Qutteina, Abdul Rahim, Hennink and Yount, 2019 [75]	Khabour, Amarneh, Bani Hani and Lataifeh, 2013 [84]	Yes	Yes	Unclear	Yes
Jones and Coast, 2013 [37]	Husain, Bevc, Husain, Chaudhry, Atif and Rahman, 2006 [40]	Yes	Yes	No	Yes
Jones and Coast, 2013 [37]	Rahman, Iqbal and Harrington, 2003 [39]	Yes	Yes	No	Yes
Jones and Coast, 2013 [37]	Rahman and Creed, 2007 [41]	Yes	Yes	No	Yes

Klainin and Arthur, 2009 [24]	Chaaya, Campbell, El Kak, Shaar, Harb and Kaddour, 2002 [25]	Yes	Yes	Unclear	Yes
Klainin and Arthur, 2009 [24]	Green, Broome and Mirabella, 2006 [26]	Yes	Yes	Unclear	Yes
Klainin and Arthur, 2009 [24]	Kalyani, Saeed, Rehman and Mubbashar, 2001 [42]	Yes	Yes	Unclear	Yes
Klainin and Arthur, 2009 [24]	Stuchbery, Matthey and Barnett, 1998 [88]	Unclear	Unclear	Unclear	Unclear
Mahendran, Puthussery and Amalan, 2019 [11]	Fareeha Hamid, 2008 [45]	Yes	Yes	No	Yes
Mahendran, Puthussery and Amalan, 2019 [11]	Ali, Azam, Ali, Tabbusum and Moin, 2012 [43]	Yes	Yes	No	Yes
Mahendran, Puthussery and Amalan, 2019 [11]	Humayun, Haider, Imran, Iqbal and Humayun, 2013 [46]	Yes	Yes	No	Yes
Mahendran, Puthussery and Amalan, 2019 [11]	Waqas, Raza, Lodhi, Muhammad, Jamal and Rehman, 2015 [52]	Yes	Yes	No	Yes
Mahendran, Puthussery and Amalan, 2019 [11]	Saeed, Raana, Saeed and Humayun, 2016 [51]	Yes	Yes	No	Yes
Mahendran, Puthussery and Amalan, 2019 [11]	Din, Ambreen, Iqbal, Iqbal and Ahmad, 2016 [44]	Yes	Yes	No	Yes
Mahendran, Puthussery and Amalan, 2019 [11]	Syeda Rabia, 2017 [49]	Yes	Yes	No	Yes
Mahendran, Puthussery and Amalan, 2019 [11]	Sharifa Mir, 2012 [48]	Yes	Yes	No	Yes
Mahendran, Puthussery and Amalan, 2019 [11]	Sadaf, 2011 [50]	Yes	Yes	No	Yes
Qutteina, Nasrallah, James-Hawkins, Nur, Yount, Hennink and Abdul Rahim, 2018 [89]	Abdelhai and Mosleh, 2015 [76]	Yes	Yes	Unclear	Yes
Qutteina, Nasrallah, James-Hawkins, Nur, Yount, Hennink and Abdul Rahim, 2018 [89]	Abou-Saleh and Ghubash, 1997 [17]	Yes	Yes	Unclear	Yes
Qutteina, Nasrallah, James-Hawkins, Nur, Yount, Hennink and Abdul Rahim, 2018 [89]	Abujilban, Abuidhail, Al-Modallal, Hamaideh and Mosemli, 2014 [78]	Yes	Yes	Unclear	Unclear
Qutteina, Nasrallah, James-Hawkins, Nur, Yount, Hennink and Abdul Rahim, 2018 [89]	Alasoom and Koura, 2014 [90]	Yes	Yes	Unclear	Yes
Qutteina, Nasrallah, James-Hawkins, Nur, Yount, Hennink and Abdul Rahim, 2018 [89]	Al-Azri, Al-Lawati, Al-Kamyani, Al-Kiyumi, Al-Rawahi, Davidson and Al-Maniri, 2016 [79]	Yes	Yes	Unclear	Unclear
Qutteina, Nasrallah, James-Hawkins, Nur, Yount, Hennink and Abdul Rahim, 2018 [89]	Bener, Burgut, Ghuloum and Sheikh, 2012 [74]	Yes	Yes	Unclear	Unclear
Qutteina, Nasrallah, James-Hawkins, Nur, Yount, Hennink and Abdul Rahim, 2018 [89]	Bener, Gerber and Sheikh, 2012 [23]	Yes	Yes	Unclear	Unclear

Qutteina, Nasrallah, James-Hawkins, Nur, Yount, Hennink and Abdul Rahim, 2018 [89]	El-Hachem, Rohayem, Bou Khalil, Richa, Kesrouani, Gemayel, Aouad, Hatab, Zaccak, Yaghi, Salameh and Attieh, 2014 [91]	Yes	Yes	Unclear	Unclear
Qutteina, Nasrallah, James-Hawkins, Nur, Yount, Hennink and Abdul Rahim, 2018 [89]	Ghubash and Abou-Saleh, 1997 [92]	Yes	Yes	Unclear	Yes
Qutteina, Nasrallah, James-Hawkins, Nur, Yount, Hennink and Abdul Rahim, 2018 [89]	Lteif, Kesrouani and Richa, 2005 [85]	Yes	Yes	Unclear	Yes
Qutteina, Nasrallah, James-Hawkins, Nur, Yount, Hennink and Abdul Rahim, 2018 [89]	Nayak and Al-Yattama, 1999 [93]	Yes	Yes	Unclear	Yes
Qutteina, Nasrallah, James-Hawkins, Nur, Yount, Hennink and Abdul Rahim, 2018 [89]	Mohammad, Gamble and Creedy, 2011 [29]	Yes	Yes	Unclear	Unclear
Qutteina, Nasrallah, James-Hawkins, Nur, Yount, Hennink and Abdul Rahim, 2018 [89]	Masmoudi, Tabelsi, Charfeddine, Ben Ayed, Guermazzi and Jaoua, 2008 [35]	Yes	Yes	Unclear	Yes
Qutteina, Nasrallah, James-Hawkins, Nur, Yount, Hennink and Abdul Rahim, 2018 [89]	Masmoudi, Trabelsi, Charfeddine, Ben Ayed, Guermazi, Jaoua, Karam and Hantouche, 2010 [87]	Yes	Yes	Unclear	Yes
Qutteina, Nasrallah, James-Hawkins, Nur, Yount, Hennink and Abdul Rahim, 2018 [89]	Masmoudi, Charfeddine, Trabelsi, Feki, Ben Ayad, Guermazi, Baati and Jaoua, 2014 [86]	Yes	Yes	Unclear	Yes
Qutteina, Nasrallah, James-Hawkins, Nur, Yount, Hennink and Abdul Rahim, 2018 [89]	Osman, Saliba, Chaaya and Naasan, 2014 [94]	Yes	Yes	Unclear	Yes
Qutteina, Nasrallah, James-Hawkins, Nur, Yount, Hennink and Abdul Rahim, 2018 [89]	Yehia, Callister and Hamdan-Mansour, 2013 [31]	Yes	Yes	Unclear	Yes
Sawyer, Ayers and Smith, 2010 [20]	Agoub, Moussaoui and Battas, 2005 [22]	Yes	Yes	No	No
Sawyer, Ayers and Smith, 2010 [20]	Alami, Kadri and Berrada, 2006 [28]	Yes	Yes	No	No
Shorey, Chee, Ng, Chan, Tam and Chong, 2018 [21]	Khalifa, Glavin, Bjertness and Lien, 2015 [54]	Yes	Yes	No	Yes
Stewart, 2007 [55]	Rahman, Lovel, Bunn, Iqbal and Harrington, 2004 [57]	Yes	Yes	No	Yes
Stewart, 2007 [55]	Rahman, Iqbal, Bunn, Lovel and Harrington, 2004 [56]	Yes	Yes	No	Yes
Zahidie and Jamali, 2013 [38]	Ali, Ali and Azam, 2009 [58]	Yes	Yes	Unclear	Yes
Zahidie and Jamali, 2013 [38]	Husain, Parveen, Husain, Saeed, Jafri, Rahman, Tomenson and Chaudhry, 2011 [47]	Yes	Yes	Unclear	Yes
Zahidie and Jamali, 2013 [38]	Asad, Karmaliani, Sullaiman, Bann, McClure, Pasha, Wright and Goldenberg, 2010 [59]	Yes	Yes	Unclear	Yes

Zahidie and Jamali, 2013 [38]	Zahidie, Kazi, Fatmi, Bhatti and Dureshahwar, 2011 [53]	Yes	Yes	Unclear	Yes

Table S8: Categorization of Risk Factors

Primary risk Category	Secondary category	Risk Factors
**Relational** Sarwar, 1990 [7, Muneer, Minhas, Tamiz-ud-Din Nizami, Mujeeb and Usmani, 2009 [10, Karmaliani, Asad, Bann, Moss, McClure, Pasha, Wright and Goldenberg, 2009 [12, Yasmeen, Tayyaba, Chatan, Naeem, Numan and Adnan Maqsood, 2010 [14, Shah, Bowen, Afridi, Nowshad and Muhajarine, 2011 [15, Abou-Saleh and Ghubash, 1997 [17, Agoub, Moussaoui and Battas, 2005 [22, Bener, Gerber and Sheikh, 2012 [23, Chaaya, Campbell, El Kak, Shaar, Harb and Kaddour, 2002 [25, Green, Broome and Mirabella, 2006 [26, Hamdan and Tamim, 2011 [27, Alami, Kadri and Berrada, 2006 [28, Mohammad, Gamble and Creedy, 2011 [29, Saleh el, El-Bahei, Del El-Hadidy and Zayed, 2013 [30, Yehia, Callister and Hamdan-Mansour, 2013 [31, Al Hinai and Al Hinai, 2014 [32, Masmoudi, Tabelsi, Charfeddine, Ben Ayed, Guermazzi and Jaoua, 2008 [35, Rahman, Iqbal and Harrington, 2003 [39, Husain, Bevc, Husain, Chaudhry, Atif and Rahman, 2006 [40, Rahman and Creed, 2007 [41, Kalyani, Saeed, Rehman and Mubbashar, 2001 [42, Ali, Ali and Azam, 2009 [58, Asad, Karmaliani, Sullaiman, Bann, McClure, Pasha, Wright and Goldenberg, 2010 [59, Gulamani, Premji, Kanji and Azam, 2013 [60, Kazi, Fatmi, Hatcher, Kadir, Niaz and Wasserman, 2006 [62, Afzal and Khalid, 2014 [69, Munaf, 2006 [71, Ghazala Sadiq, 2015 [72, Al Dallal and Grant, 2012 [73, Bener, Burgut, Ghuloum and Sheikh, 2012 [74, Abdelhai and Mosleh, 2015 [76, Abujilban, Abuidhail, Al-Modallal, Hamaideh and Mosemli, 2014 [78, Al-Azri, Al-Lawati, Al-Kamyani, Al-Kiyumi, Al-Rawahi, Davidson and Al-Maniri, 2016 [79, Lteif, Kesrouani and Richa, 2005 [85, Masmoudi, Trabelsi, Charfeddine, Ben Ayed, Guermazi, Jaoua, Karam and Hantouche, 2010 [87, Alasoom and Koura, 2014 [90, El-Hachem, Rohayem, Bou Khalil, Richa, Kesrouani, Gemayel, Aouad, Hatab, Zaccak, Yaghi, Salameh and Attieh, 2014 [91, Nayak and Al-Yattama, 1999 [93, Osman, Saliba, Chaaya and Naasan, 2014 [94, Amr and Hussein Balaha, 2010 [95, Ghubash and Eapen, 2009 [96]	**Spousal relationships**	Domestic violence, Assault history (familial or non-familial), Verbal or physical/ sexual abuse, Marital difficulty, Relationship with spouse, Satisfaction in married life, Spousal support, Partner support, Sexual relationship prior to pregnancy, Sexual relationship during pregnancy, Husband away, Polygamy
	**Parent/ caregiver-child relationships**	Unplanned pregnancy, Number of living children, Parity, Ability to carry out daily activities
	**Parental physical or mental health**	
	**Social and peer supports**	Social support, Traditional practice (chilla period), Religious practices, Help at home /Family support, Type of family, Maternal help, Paternal presence, Confidants or friends, Relationship with mother-in-law, Familial relationships, Familial conflicts, Sick relatives, Alcoholism in family, self-image, Self-esteem and self-efficacy
	**Community environment**	Sex of the baby
**Psychological** Sarwar, 1990 [7, Muneer, Minhas, Tamiz-ud-Din Nizami, Mujeeb and Usmani, 2009 [10, Karmaliani, Asad, Bann, Moss, McClure, Pasha, Wright and Goldenberg, 2009 [12, Yasmeen, Tayyaba, Chatan,	**Psychosocial vulnerabilities and strengths**	Financial independence of the woman, Education levels (Low and High VS med, Secondary or low VS University, Literate VS

Naeem, Numan and Adnan Maqsood, 2010 [14, Agoub, Moussaoui and Battas, 2005 [22, Bener, Gerber and Sheikh, 2012 [23, Chaaya, Campbell, El Kak, Shaar, Harb and Kaddour, 2002 [25, Green, Broome and Mirabella, 2006 [26, Hamdan and Tamim, 2011 [27, Alami, Kadri and Berrada, 2006 [28, Mohammad, Gamble and Creedy, 2011 [29, Masmoudi, Tabelsi, Charfeddine, Ben Ayed, Guermazzi and Jaoua, 2008 [35, Rahman, Iqbal and Harrington, 2003 [39, Husain, Bevc, Husain, Chaudhry, Atif and Rahman, 2006 [40, Rahman and Creed, 2007 [41, Al Dallal and Grant, 2012 [73, Bener, Burgut, Ghuloum and Sheikh, 2012 [74, Abuidhail and Abujilban, 2014 [77, Abujilban, Abuidhail, Al-Modallal, Hamaideh and Mosemli, 2014 [78, Al-Azri, Al-Lawati, Al-Kamyani, Al-Kiyumi, Al-Rawahi, Davidson and Al-Maniri, 2016 [79, Alharbi and Abdulghani, 2014 [80, Bener, 2013 [81, Burgut, Bener, Ghuloum and Sheikh, 2013 [82, El-Khoury, Karam and Melhem, 1999 [83, Khabour, Amarneh, Bani Hani and Lataifeh, 2013 [84, Masmoudi, Charfeddine, Trabelsi, Feki, Ben Ayad, Guermazi, Baati and Jaoua, 2014 [86, Masmoudi, Trabelsi, Charfeddine, Ben Ayed, Guermazi, Jaoua, Karam and Hantouche, 2010 [87]		illiterate), Parenting knowledge/ skills, Employment status VS working)
	**Neuropsychological and neurodevelopmental indicators**	
	**Mental health history**	Antenatal depression, Postpartum blues, Mental illness in the antenatal period
**Physiological and health** Haider, 2010 [13, Agoub, Moussaoui and Battas, 2005 [22, Chaaya, Campbell, El Kak, Shaar, Harb and Kaddour, 2002 [25, Green, Broome and Mirabella, 2006 [26, Hamdan and Tamim, 2011 [27, Alami, Kadri and Berrada, 2006 [28, Saleh el, El-Bahei, Del El-Hadidy and Zayed, 2013 [30, Yehia, Callister and Hamdan-Mansour, 2013 [31, Rahman, Iqbal and Harrington, 2003 [39, Husain, Parveen, Husain, Saeed, Jafri, Rahman, Tomenson and Chaudhry, 2011 [47, Ali, Ali and Azam, 2009 [58, Gulamani, Premji, Kanji and Azam, 2013 [60, Kazi, Fatmi, Hatcher, Kadir, Niaz and Wasserman, 2006 [62, Amr and Hussein Balaha, 2010 [95, Ghubash and Eapen, 2009 [96]	**Physiological vulnerabilities and strengths**	Disability, Chronic health problems
	**Obstetric and perinatal factors**	Difficulty in delivery, Mode of delivery (Caesarean delivery VS Vaginal delivery), Previous miscarriage/ difficult pregnancies, Complications during pregnancy, Anaemia, Difficulty in breast feeding, Baby's health problems (cyanosis/ apnoea/flaccidity), Preterm infants, Concerns about well-being of unborn baby,
**Occupational** Al Hinai and Al Hinai, 2014 [32, Lteif, Kesrouani and Richa, 2005 [85]	**Work environment**	Work difficulties
	**Employee health**	
	**High risk occupations/ roles**	
**Sociodemographic** Muneer, Minhas, Tamiz-ud-Din Nizami, Mujeeb and Usmani, 2009 [10, Karmaliani, Asad, Bann, Moss, McClure, Pasha, Wright	**Non-modifiable sociodemographic factors**	Age, Age at marriage, Duration of marriage

and Goldenberg, 2009 [12, Yasmeen, Tayyaba, Chatan, Naeem, Numan and Adnan Maqsood, 2010 [14, Agoub, Moussaoui and Battas, 2005 [22, Bener, Gerber and Sheikh, 2012 [23, Chaaya, Campbell, El Kak, Shaar, Harb and Kaddour, 2002 [25, Green, Broome and Mirabella, 2006 [26, Hamdan and Tamim, 2011 [27, Alami, Kadri and Berrada, 2006 [28, Mohammad, Gamble and Creedy, 2011 [29, Saleh el, El-Bahei, Del El-Hadidy and Zayed, 2013 [30, Yehia, Callister and Hamdan-Mansour, 2013 [31, Al Hinai and Al Hinai, 2014 [32, Naglaa A. Mohamed and Maklof, 2011 [33, Rahman, Iqbal and Harrington, 2003 [39, Rahman and Creed, 2007 [41, Zahidie, Kazi, Fatmi, Bhatti and Dureshahwar, 2011 [53, Kazi, Fatmi, Hatcher, Kadir, Niaz and Wasserman, 2006 [62, Ghazala Sadiq, 2015 [72, Al Dallal and Grant, 2012 [73, Bener, Burgut, Ghuloum and Sheikh, 2012 [74, Abdelhai and Mosleh, 2015 [76, Al-Azri, Al-Lawati, Al-Kamyani, Al-Kiyumi, Al-Rawahi, Davidson and Al-Maniri, 2016 [79, Alharbi and Abdulghani, 2014 [80, Burgut, Bener, Ghuloum and Sheikh, 2013 [82, Khabour, Amarneh, Bani Hani and Lataifeh, 2013 [84, Amr and Hussein Balaha, 2010 [95]	**Modifiable sociodemographic factors**	Husband's monthly income, Husband's employment, Family wealth, Financial distress, Satisfaction with income, Husband's education levels, Place of residence (Urban VS Rural)
**Predictors of response to trauma**<br>Sarwar, 1990 [7, Haider, 2010 [13, Abou-Saleh and Ghubash, 1997 [17, Agoub, Moussaoui and Battas, 2005 [22, Chaaya, Campbell, El Kak, Shaar, Harb and Kaddour, 2002 [25, Hamdan and Tamim, 2011 [27, Alami, Kadri and Berrada, 2006 [28, Balaha, Amr, El-Gilany and Sheikh, 2009 [34, Rahman, Iqbal and Harrington, 2003 [39, Husain, Bevc, Husain, Chaudhry, Atif and Rahman, 2006 [40, Kalyani, Saeed, Rehman and Mubbashar, 2001 [42, Kazi, Fatmi, Hatcher, Kadir, Niaz and Wasserman, 2006 [62, Amr and Hussein Balaha, 2010 [95]; Saleh el, El-Bahei, Del El-Hadidy and Zayed, 2013 [30, Naglaa A. Mohamed and Maklof, 2011 [33, Husain, Parveen, Husain, Saeed, Jafri, Rahman, Tomenson and Chaudhry, 2011 [47]		Stressful life events in the preceding year, Threatening life events, Early loss of mother, Psychiatric history, Psychiatric disorders
**Lifestyle**	**Diet**	No study reported these risk factors
	**Physical activity**	
	**Risk behaviour**	
	**Mental activity**	
**Negative environmental exposures**	**Chemical and physical exposures**	
	**Media exposures**	
**Genetic**		

	Neuroanatomical/ neurochemical

References

1. Moher, D., A. Liberati, J. Tetzlaff, and D. G. Altman. "Preferred Reporting Items for Systematic Reviews and Meta-Analyses: The Prisma Statement." *J Clin Epidemiol* 62, no. 10 (2009): 1006-12.
2. Zorzela, Liliane, Yoon K Loke, John P Ioannidis, Su Golder, Pasqualina Santaguida, Douglas G Altman, David Moher, and Sunita Vohra. "Prisma Harms Checklist: Improving Harms Reporting in Systematic Reviews." *Bmj* 352 (2016): i157.
3. Beller, E. M., P. P. Glasziou, D. G. Altman, S. Hopewell, H. Bastian, I. Chalmers, P. C. Gotzsche, T. Lasserson, and D. Tovey. "Prisma for Abstracts: Reporting Systematic Reviews in Journal and Conference Abstracts." *PLoS Med* 10, no. 4 (2013): e1001419.
4. Afzal, Sidra, and Ruhi Khalid. "Postnatal Affective Disorders: Mapping the Research in Pakistan." (2016).
5. Rasheed, H., & Khalid, R. . "Maternity Blues and Symptoms of Puerperal Depression Four Weeks after Child Birth: A Comparitive Study. ." (1988).
6. Khalid, Ruhi. "Maternity Blues and Puerperal Depression in Pakistani Women." *Pakistan Journal of Psychological Research* 4 (1989): 3-4.
7. Sarwar, A., & Khalid, R. "To Stuady Postnatal Depression among Pakistani Women." (1990).
8. Habib, S., & Khalid, R. . "Pregnancy Related Psychiatric Problems." (1997).
9. Ahmed, Maria khan and Iftikhar. "Risk Factors Associated with Postnatal Depression in Pakistani Women." *Pakistan Journal of Social and Clinical Psychology* 3 (2005): 41-50.
10. Muneer, A., F. A. Minhas, A. Tamiz-ud-Din Nizami, F. Mujeeb, and A. T. Usmani. "Frequency and Associated Factors for Postnatal Depression." *Journal of the College of Physicians and Surgeons--Pakistan* 19, no. 4 (2009): 236-9.
11. Mahendran, R., S. Puthussery, and M. Amalan. "Prevalence of Antenatal Depression in South Asia: A Systematic Review and Meta-Analysis." *Journal of Epidemiology and Community Health* 73, no. 8 (2019): 768-77.
12. Karmaliani, R., N. Asad, C. M. Bann, N. Moss, E. M. McClure, O. Pasha, L. L. Wright, and R. L. Goldenberg. "Prevalence of Anxiety, Depression and Associated Factors among Pregnant Women of Hyderabad, Pakistan." *International Journal of Social Psychiatry* 55, no. 5 (2009): 414-24.
13. Haider, Imran &. "Screening of Antenatal Depression in Pakistan: Risk Factors and Effects on Obsetric and Neonatal Outcomes." *Asia-Paciifc Psychiatry* 2 (2010): 26-32.
14. Yasmeen, Khooharo, Majeed Tayyaba, Das Chatan, Majeed Naeem, Majeed Numan, and Choudhry Adnan Maqsood. "Associated Risk Factors for Postpartum Depression Presenting at a Teaching Hospital." *Annals of King Edward Medical University* 16, no. 2 (2010).
15. Shah, S. M., A. Bowen, I. Afridi, G. Nowshad, and N. Muhajarine. "Prevalence of Antenatal Depression: Comparison between Pakistani and Canadian Women." *Journal of the Pakistan Medical Association* 61, no. 3 (2011): 242-6.
16. Alhasanat, Dalia, and Judith Fry-McComish. "Postpartum Depression among Immigrant and Arabic Women: Literature Review." *Journal of Immigrant and Minority Health* 17, no. 6 (2015): 1882-94.
17. Abou-Saleh, M. T., and R. Ghubash. "The Prevalence of Early Postpartum Psychiatric Morbidity in Dubai: A Transcultural Perspective." *Acta Psychiatrica Scandinavica* 95, no. 5 (1997): 428-32.
18. Amber Haque, Azimeh Namavar, Kelly-Ann Breene. "Prevalence and Risk Factors of Postpartum Depression in Middle Eastern/Arab Women." *Journal of Muslim Mental Health* 9, no. 1 (2015).
19. Fisher, J., M. Cabral de Mello, V. Patel, A. Rahman, T. Tran, S. Holton, and W. Holmes. "Prevalence and Determinants of Common Perinatal Mental Disorders in Women in Low- and Lower-Middle-Income Countries: A Systematic Review." *Bull World Health Organ* 90, no. 2 (2012): 139g-49g.
20. Sawyer, A., S. Ayers, and H. Smith. "Pre- and Postnatal Psychological Wellbeing in Africa: A Systematic Review." *Journal of Affective Disorders* 123, no. 1-3 (2010): 17-29.

21. Shorey, S., C. Y. I. Chee, E. D. Ng, Y. H. Chan, W. W. S. Tam, and Y. S. Chong. "Prevalence and Incidence of Postpartum Depression among Healthy Mothers: A Systematic Review and Meta-Analysis." *Journal of Psychiatric Research* 104 (2018): 235-48.
22. Agoub, M., D. Moussaoui, and O. Battas. "Prevalence of Postpartum Depression in a Moroccan Sample." *Arch Womens Ment Health* 8, no. 1 (2005): 37-43.
23. Bener, A., L. M. Gerber, and J. Sheikh. "Prevalence of Psychiatric Disorders and Associated Risk Factors in Women During Their Postpartum Period: A Major Public Health Problem and Global Comparison." *Int J Womens Health* 4 (2012): 191-200.
24. Klainin, P., and D. G. Arthur. "Postpartum Depression in Asian Cultures: A Literature Review." *International Journal of Nursing Studies* 46, no. 10 (2009): 1355-73.
25. Chaaya, M., O. M. Campbell, F. El Kak, D. Shaar, H. Harb, and A. Kaddour. "Postpartum Depression: Prevalence and Determinants in Lebanon." *Arch Womens Ment Health* 5, no. 2 (2002): 65-72.
26. Green, K., H. Broome, and J. Mirabella. "Postnatal Depression among Mothers in the United Arab Emirates: Socio-Cultural and Physical Factors." *Psychology, Health & Medicine* 11, no. 4 (2006): 425-31.
27. Hamdan, A., and H. Tamim. "Psychosocial Risk and Protective Factors for Postpartum Depression in the United Arab Emirates." *Arch Womens Ment Health* 14, no. 2 (2011): 125-33.
28. Alami, K. M., N. Kadri, and S. Berrada. "Prevalence and Psychosocial Correlates of Depressed Mood During Pregnancy and after Childbirth in a Moroccan Sample." *Arch Womens Ment Health* 9, no. 6 (2006): 343-6.
29. Mohammad, K. I., J. Gamble, and D. K. Creedy. "Prevalence and Factors Associated with the Development of Antenatal and Postnatal Depression among Jordanian Women." *Midwifery* 27, no. 6 (2011): e238-45.
30. Saleh el, S., W. El-Bahei, M. A. Del El-Hadidy, and A. Zayed. "Predictors of Postpartum Depression in a Sample of Egyptian Women." *Neuropsychiatric Disease and Treatment* 9 (2013): 15-24.
31. Yehia, D. B., L. C. Callister, and A. Hamdan-Mansour. "Prevalence and Predictors of Postpartum Depression among Arabic Muslim Jordanian Women Serving in the Military." *Journal of Perinatal and Neonatal Nursing* 27, no. 1 (2013): 25-33; quiz 34-5.
32. Al Hinai, F. I., and S. S. Al Hinai. "Prospective Study on Prevalence and Risk Factors of Postpartum Depression in Al-Dakhliya Governorate in Oman." *Oman Medical Journal* 29, no. 3 (2014): 198-202.
33. Naglaa A. Mohamed, Ghadah A. Mahmoud, Nadia A. Said, K Hamida A. Abdelhafez , and and Ahmed M. A. Maklof. "Postpartum Depression: Prevalence and Predictors among Women at El Eman's Specialized Hospital " *Journal of American Science* 7 (12) (2011): 122-28.
34. Balaha, Magdy, Mostafa Amr, Abdel-Hady El-Gilany, and Farid Sheikh. "Obstetric and Psychiatric Outcomes in a Sample of Saudi Teen-Aged Mothers." *TAF Preventive Medicine Bulletin* 8 (2009).
35. Masmoudi, J., S. Tabelsi, F. Charfeddine, B. Ben Ayed, M. Guermazzi, and A. Jaoua. "[Study of the Prevalence of Postpartum Depression among 213 Tunisian Parturients]." *Gynécologie, Obstétrique & Fertilité* 36, no. 7-8 (2008): 782-7.
36. Karmaliani, R., C. M. Bann, M. A. Mahmood, H. S. Harris, S. Akhtar, R. L. Goldenberg, and N. Moss. "Measuring Antenatal Depression and Anxiety: Findings from a Community-Based Study of Women in Hyderabad, Pakistan." *Women and Health* 44, no. 3 (2006): 79-103.
37. Jones, E., and E. Coast. "Social Relationships and Postpartum Depression in South Asia: A Systematic Review." *International Journal of Social Psychiatry* 59, no. 7 (2013): 690-700.
38. Zahidie, A., and T. Jamali. "An Overview of the Predictors of Depression among Adult Pakistani Women." *Journal of the College of Physicians and Surgeons--Pakistan* 23, no. 8 (2013): 574-80.
39. Rahman, A., Z. Iqbal, and R. Harrington. "Life Events, Social Support and Depression in Childbirth: Perspectives from a Rural Community in the Developing World." *Psychological Medicine* 33, no. 7 (2003): 1161-7.

40. Husain, N., I. Bevc, M. Husain, I. B. Chaudhry, N. Atif, and A. Rahman. "Prevalence and Social Correlates of Postnatal Depression in a Low Income Country." *Arch Womens Ment Health* 9, no. 4 (2006): 197-202.
41. Rahman, A., and F. Creed. "Outcome of Prenatal Depression and Risk Factors Associated with Persistence in the First Postnatal Year: Prospective Study from Rawalpindi, Pakistan." *Journal of Affective Disorders* 100, no. 1-3 (2007): 115-21.
42. Kalyani, G. H. S., Khitam Saeed, Ch I. U. Rehman, and M. H. Mubbashar. "Incidence of Depressive Illness in Pakistani Women During Postnatal Period." *Journal of the College of Physicians and Surgeons Pakistan* 11(4) (2001): 246-48.
43. Ali, N. S., I. S. Azam, B. S. Ali, G. Tabbusum, and S. S. Moin. "Frequency and Associated Factors for Anxiety and Depression in Pregnant Women: A Hospital-Based Cross-Sectional Study." *ScientificWorldJournal* 2012 (2012): 653098.
44. Din, Z. U., S. Ambreen, Z. Iqbal, M. Iqbal, and S. Ahmad. "Determinants of Antenatal Psychological Distress in Pakistani Women." *Noro Psikiyatr Ars* 53, no. 2 (2016): 152-57.
45. Fareeha Hamid, Aftab Asif, Imran Ijaz Haider. "Study of Anxiety and Depression During Pregnancy." *Pakistan Journal of Medical Sciences* 24 (2008): 861-64.
46. Humayun, A., Haider, II, N. Imran, H. Iqbal, and N. Humayun. "Antenatal Depression and Its Predictors in Lahore, Pakistan." *East Mediterr Health J* 19, no. 4 (2013): 327-32.
47. Husain, N., A. Parveen, M. Husain, Q. Saeed, F. Jafri, R. Rahman, B. Tomenson, and I. B. Chaudhry. "Prevalence and Psychosocial Correlates of Perinatal Depression: A Cohort Study from Urban Pakistan." *Arch Womens Ment Health* 14, no. 5 (2011): 395-403.
48. Sharifa Mir, Rozina Karmaliani, Juanita Hatcher, Nargis Asad, Siham Sikander. "Prevalence and Risk Factors Contributing to Depression among Pregnant Women in District Chitral, Pakistan " *Journal of Pakistani PsychiatricSociety* 9 (2012): 28-36.
49. Syeda Rabia, Uzma Nusrat, Sara Qazi, Hira Afreen. "Frequency and Risk Profiles Associated with Antenatal Anxiety and Depression in Middle Socioeconomic Women." *Annals Abbasi Shaheed Hospital & Karachi Medical & Dental College* 22 (2017): 88-96.
50. Sadaf, M. "Antenatal Screening for Postpartum Depression." *Journal of Rawalpindi Medical College* 15 (2011): 47-49.
51. Saeed, Ayesha, Tahira Raana, Amina Muhammad Saeed, and Ayesha Humayun. "Effect of Antenatal Depression on Maternal Dietary Intake and Neonatal Outcome: A Prospective Cohort." *Nutrition Journal* 15, no. 1 (2016): 64-64.
52. Waqas, A., N. Raza, H. W. Lodhi, Z. Muhammad, M. Jamal, and A. Rehman. "Psychosocial Factors of Antenatal Anxiety and Depression in Pakistan: Is Social Support a Mediator?" *PloS One* 10, no. 1 (2015): e0116510.
53. Zahidie, A., A. Kazi, Z. Fatmi, M. T. Bhatti, and S. Dureshahwar. "Social Environment and Depression among Pregnant Women in Rural Areas of Sind, Pakistan." *Journal of the Pakistan Medical Association* 61, no. 12 (2011): 1183-9.
54. Khalifa, D. S., K. Glavin, E. Bjertness, and L. Lien. "Postnatal Depression among Sudanese Women: Prevalence and Validation of the Edinburgh Postnatal Depression Scale at 3 Months Postpartum." *Int J Womens Health* 7 (2015): 677-84.
55. Stewart, R. C. "Maternal Depression and Infant Growth: A Review of Recent Evidence." *Maternal & Child Nutrition* 3, no. 2 (2007): 94-107.
56. Rahman, A., Z. Iqbal, J. Bunn, H. Lovel, and R. Harrington. "Impact of Maternal Depression on Infant Nutritional Status and Illness: A Cohort Study." *Archives of General Psychiatry* 61, no. 9 (2004): 946-52.
57. Rahman, A., H. Lovel, J. Bunn, Z. Iqbal, and R. Harrington. "Mothers' Mental Health and Infant Growth: A Case-Control Study from Rawalpindi, Pakistan." *Child: Care, Health and Development* 30, no. 1 (2004): 21-7.
58. Ali, N. S., B. S. Ali, and I. S. Azam. "Post Partum Anxiety and Depression in Peri-Urban Communities of Karachi, Pakistan: A Quasi-Experimental Study." *BMC Public Health* 9 (2009): 384.
59. Asad, N., R. Karmaliani, N. Sullaiman, C. M. Bann, E. M. McClure, O. Pasha, L. L. Wright, and R. L. Goldenberg. "Prevalence of Suicidal Thoughts and Attempts among Pregnant Pakistani Women." *Acta Obstetricia et Gynecologica Scandinavica* 89, no. 12 (2010): 1545-51.

60. Gulamani, Salima S., Shahirose Sadrudin Premji, ZeenatKhanu Kanji, and Syed Iqbal Azam. "A Review of Postpartum Depression, Preterm Birth, and Culture." *The Journal of Perinatal & Neonatal Nursing* 27, no. 1 (2013): 52-59.
61. Husain, Nusrat, Tariq Munshi, Farhat Jafri, Meher Husain, Asia Parveen, Qamar Saeed, Barbara Tomenson, Farooq Naeem, and Nasim Chaudhry. "Antenatal Depression Is Not Associated with Low-Birth Weight: A Study from Urban Pakistan." *Frontiers in Psychiatry* 5 (2014).
62. Kazi, Ambreen, Zafar Fatmi, Juanita Hatcher, Muhammad Masood Kadir, Unaiza Niaz, and Gail A. Wasserman. "Social Environment and Depression among Pregnant Women in Urban Areas of Pakistan: Importance of Social Relations." *Social Science & Medicine* 63, no. 6 (2006): 1466-76.
63. Al-Modayfer, Omar, Yousra Alatiq, Osaima Khair, and Sami Abdelkawi. "Postpartum Depression and Related Risk Factors among Saudi Females." *International Journal of Culture and Mental Health* 8, no. 3 (2015): 316-24.
64. Fuhr, D. C., C. Calvert, C. Ronsmans, P. S. Chandra, S. Sikander, M. J. De Silva, and V. Patel. "Contribution of Suicide and Injuries to Pregnancy-Related Mortality in Low-Income and Middle-Income Countries: A Systematic Review and Meta-Analysis." *Lancet Psychiatry* 1, no. 3 (2014): 213-25.
65. Farhat, E. B., M. Chaouch, H. Chelli, M. F. Gara, N. Boukraa, M. Garbouj, M. Hamrouni, A. Fourati, T. Calvez, and P. Thonneau. "Reduced Maternal Mortality in Tunisia and Voluntary Commitment to Gender-Related Concerns." *International Journal of Gynaecology and Obstetrics* 116, no. 2 (2012): 165-8.
66. Amarin, Z., Y. Khader, A. Okour, H. Jaddou, and R. Al-Qutob. "National Maternal Mortality Ratio for Jordan, 2007-2008." *International Journal of Gynaecology and Obstetrics* 111, no. 2 (2010): 152-6.
67. Jafarey, S. N., T. Rizvi, M. Koblinsky, and N. Kureshy. "Verbal Autopsy of Maternal Deaths in Two Districts of Pakistan--Filling Information Gaps." *Journal of Health, Population, and Nutrition* 27, no. 2 (2009): 170-83.
68. Population, Ministry of Health and. "The National Maternal Mortality Study:Egypt 2000." Egypt, 2001.
69. Afzal, Sidra, and Ruhi Khalid. "Social Support and Postnatal Depression in Pakistani Context." *Pakistan Journal of Social and Clinical Psychology* 12 (2014): 34-38.
70. Gul, Mirrat, Saqib Bajwa, Saima Niaz, Mariam Haroon, Saima Liaqat, Mafia Ahmad, Sana Dawood, Fiza Ghazal, Ali Abbass Bhatti, Sadia Nazir, Syeda Rabia Riffat, and Haroon Rashid Chaudhry. "Postnatal Depression and Its Comparison with the Gender of Newborn in Fourth Pregnancy." *International Journal of Culture and Mental Health* 6, no. 1 (2013): 16-28.
71. Munaf, Riaz ahmed and Seema. "Nuclear Family System as an Important Risk Factor for the Development of the Baby Blues." *Pakistan Journal of Social and Clinical Psychology* 4 (2006): 67-74.
72. Ghazala Sadiq, Shahzad Sadiq, Namrah Ali. "Evaluation of Postpartum Depression in Rawalpindi/ Islamabad Pakistan." *EC Gynaecology* 1.1 (2015): 19-25.
73. Al Dallal, F. H., and I. N. Grant. "Postnatal Depression among Bahraini Women: Prevalence of Symptoms and Psychosocial Risk Factors." *East Mediterr Health J* 18, no. 5 (2012): 439-45.
74. Bener, A., F. T. Burgut, S. Ghuloum, and J. Sheikh. "A Study of Postpartum Depression in a Fast Developing Country: Prevalence and Related Factors." *International Journal of Psychiatry in Medicine* 43, no. 4 (2012): 325-37.
75. James-Hawkins, L., E. Shaltout, A. A. Nur, C. Nasrallah, Y. Qutteina, H. F. Abdul Rahim, M. Hennink, and K. M. Yount. "Human and Economic Resources for Empowerment and Pregnancy-Related Mental Health in the Arab Middle East: A Systematic Review." *Arch Womens Ment Health* 22, no. 1 (2019): 1-14.
76. Abdelhai, R., and H. Mosleh. "Screening for Antepartum Anxiety and Depression and Their Association with Domestic Violence among Egyptian Pregnant Women." *Journal of the Egyptian Public Health Association* 90, no. 3 (2015): 101-8.
77. Abuidhail, J., and S. Abujilban. "Characteristics of Jordanian Depressed Pregnant Women: A Comparison Study." *Journal of Psychiatric and Mental Health Nursing* 21, no. 7 (2014): 573-9.
78. Abujilban, S. K., J. Abuidhail, H. Al-Modallal, S. Hamaideh, and O. Mosemli. "Predictors of Antenatal Depression among Jordanian Pregnant Women in Their Third Trimester." *Health Care for Women International* 35, no. 2 (2014): 200-15.

79. Al-Azri, M., I. Al-Lawati, R. Al-Kamyani, M. Al-Kiyumi, A. Al-Rawahi, R. Davidson, and A. Al-Maniri. "Prevalence and Risk Factors of Antenatal Depression among Omani Women in a Primary Care Setting: Cross-Sectional Study." *Sultan Qaboos University Medical Journal* 16, no. 1 (2016): e35-41.
80. Alharbi, A. A., and H. M. Abdulghani. "Risk Factors Associated with Postpartum Depression in the Saudi Population." *Neuropsychiatric Disease and Treatment* 10 (2014): 311-6.
81. Bener, A. "Psychological Distress among Postpartum Mothers of Preterm Infants and Associated Factors: A Neglected Public Health Problem." *Braz J Psychiatry* 35, no. 3 (2013): 231-6.
82. Burgut, F. T., A. Bener, S. Ghuloum, and J. Sheikh. "A Study of Postpartum Depression and Maternal Risk Factors in Qatar." *Journal of Psychosomatic Obstetrics and Gynaecology* 34, no. 2 (2013): 90-7.
83. El-Khoury, N., E. G. Karam, and N. M. Melhem. "[Depression and Pregnancy]." *Journal Medical Libanais. Lebanese Medical Journal* 47, no. 3 (1999): 169-74.
84. Khabour, O., B. Amarneh, E. Bani Hani, and I. Lataifeh. "Associations between Variations in Tph1 , Tph2 and Slc6a4 Genes and Postpartum Depression: A Study in the Jordanian Population." *Balkan Journal of Medical Genetics* 16, no. 1 (2013): 41-8.
85. Lteif, Y., A. Kesrouani, and S. Richa. "[Depressive Syndromes During Pregnancy: Prevalence and Risk Factors]." *Journal de Gynécologie, Obstétrique et Biologie de la Reproduction* 34, no. 3 Pt 1 (2005): 262-9.
86. Masmoudi, J., F. Charfeddine, S. Trabelsi, I. Feki, B. Ben Ayad, M. Guermazi, I. Baati, and A. Jaoua. "[Postpartum Depression: Prevalence and Risk Factors. A Prospective Study Concerning 302 Tunisian Parturients]." *Tunisie Medicale* 92, no. 10 (2014): 615-21.
87. Masmoudi, J., S. Trabelsi, F. Charfeddine, B. Ben Ayed, M. Guermazi, A. Jaoua, E. G. Karam, and E. G. Hantouche. "[Evaluation of Affective Temperaments in the Postpartum Depressive Symptomatology]." *Encephale* 36 Suppl 2 (2010): D14-21.
88. Stuchbery, M., S. Matthey, and B. Barnett. "Postnatal Depression and Social Supports in Vietnamese, Arabic and Anglo-Celtic Mothers." *Social Psychiatry and Psychiatric Epidemiology* 33, no. 10 (1998): 483-90.
89. Qutteina, Y., C. Nasrallah, L. James-Hawkins, A. A. Nur, K. M. Yount, M. Hennink, and H. F. Abdul Rahim. "Social Resources and Arab Women's Perinatal Mental Health: A Systematic Review." *Women Birth* 31, no. 5 (2018): 386-97.
90. Alasoom, L. I., and M. R. Koura. "Predictors of Postpartum Depression in the Eastern Province Capital of Saudi Arabia." *J Family Med Prim Care* 3, no. 2 (2014): 146-50.
91. El-Hachem, C., J. Rohayem, R. Bou Khalil, S. Richa, A. Kesrouani, R. Gemayel, N. Aouad, N. Hatab, E. Zaccak, N. Yaghi, S. Salameh, and E. Attieh. "Early Identification of Women at Risk of Postpartum Depression Using the Edinburgh Postnatal Depression Scale (Epds) in a Sample of Lebanese Women." *BMC Psychiatry* 14 (2014): 242.
92. Ghubash, R., and M. T. Abou-Saleh. "Postpartum Psychiatric Illness in Arab Culture: Prevalence and Psychosocial Correlates." *British Journal of Psychiatry* 171 (1997): 65-8.
93. Nayak, M. B., and M. Al-Yattama. "Assault Victim History as a Factor in Depression During Pregnancy." *Obstetrics and Gynecology* 94, no. 2 (1999): 204-8.
94. Osman, H., M. Saliba, M. Chaaya, and G. Naasan. "Interventions to Reduce Postpartum Stress in First-Time Mothers: A Randomized-Controlled Trial." *BMC Women's Health* 14 (2014): 125.
95. Amr, M. A., and M. H. Hussein Balaha. "Minor Psychiatric Morbidity in Young Saudi Mothers Using Mini International Neuropsychiatric Interview (Mini)." *Journal of the College of Physicians and Surgeons--Pakistan* 20, no. 10 (2010): 680-4.
96. Ghubash, R., and V. Eapen. "Postpartum Mental Illness: Perspectives from an Arabian Gulf Population." *Psychological Reports* 105, no. 1 (2009): 127-36.
